# Catalase Activity and Methylglyoxal Concentration in Plasma and Erythrocytes Across Different Clinical Phenotypes of Thyroid Disease: A Cross-Sectional Study

**DOI:** 10.3390/diseases14100357

**Published:** 2026-09-26

**Authors:** Ernur Bekov, Olga Ponamareva, Ryszhan Bakirova, Vilen Molotov-Luchanskiy, Yelena Pozdnyakova

**Affiliations:** 1 Department of Internal Medicine, Karaganda Medical University, Karaganda 100008, Kazakhstan; 2 Department of Biomedicine, Karaganda Medical University, Karaganda 100008, Kazakhstan

**Keywords:** catalase, methylglyoxal, autoimmune thyroiditis, hypothyroidism, euthyroidism, nontoxic nodular goiter, erythrocytes, plasma

## Abstract

**Background/Objectives:** Thyroid disorders may involve systemic oxidative and carbonyl disturbances, but compartment-specific changes in catalase activity and methylglyoxal concentration across different clinical phenotypes remain unclear. This study aimed to evaluate catalase activity and methylglyoxal concentrations in erythrocytes and plasma across predefined clinical phenotypes representing different combinations of thyroid functional, autoimmune, and structural characteristics and to examine correlations among these variables within and between the erythrocyte and plasma compartments. **Methods:** This single-center cross-sectional study included 164 participants: 24 controls, 31 patients with hypothyroidism without autoimmune thyroiditis, 30 with autoimmune thyroiditis and hypothyroidism, 42 with euthyroid nontoxic nodular goiter, and 37 with euthyroid autoimmune thyroiditis. Catalase activity and methylglyoxal concentration were measured spectrophotometrically. **Results:** Erythrocyte catalase activity was higher in all patient groups than in controls. Plasma catalase activity was significantly higher in hypothyroidism without autoimmune thyroiditis and nontoxic nodular goiter, but not in autoimmune thyroiditis with hypothyroidism or euthyroid autoimmune thyroiditis. Erythrocyte MGO concentration was significantly lower in hypothyroidism without autoimmune thyroiditis and nontoxic nodular goiter, whereas autoimmune thyroiditis with hypothyroidism and euthyroid autoimmune thyroiditis did not differ significantly from controls. Plasma MGO concentration did not differ significantly among groups. In patients, catalase activity was not correlated with MGO within either compartment, whereas erythrocyte and plasma MGO concentrations were positively correlated. **Conclusions:** Thyroid disorders showed nonuniform group-level patterns of catalase activity and MGO concentration across the erythrocyte and plasma compartments. The findings support separate interpretation of erythrocyte and plasma measurements but do not establish distinct phenotype-specific biomarker profiles.

## 1. Introduction

Hashimoto thyroiditis (HT) is one of the most prevalent autoimmune thyroid disorders and a leading cause of primary hypothyroidism in iodine-sufficient regions [1]. The clinical heterogeneity of HT is reflected in the fact that some patients develop hypothyroidism as a consequence of immune-mediated thyroid injury, whereas others remain euthyroid despite the presence of thyroid autoantibodies and ultrasonographic features of chronic thyroiditis [2,3]. Accordingly, studies of systemic biochemical abnormalities in thyroid disease should distinguish not only between hypothyroid and euthyroid patients but also between patients with and without an autoimmune component, because these phenotypes may represent different combinations of thyroid hormone deficiency, immune-mediated inflammation, and tissue injury [4].

A distinctive feature of the thyroid gland is that thyroid hormone biosynthesis inherently depends on oxidative reactions. The iodination of thyroglobulin and the synthesis of thyroid hormones require thyroid peroxidase and hydrogen peroxide, making thyroid tissue particularly dependent on tight regulation of the balance between H_2_O_2_ generation and elimination [5,6].

Hydrogen peroxide in the thyroid gland is not only an essential substrate for thyroid hormone biosynthesis but also a potential mediator of tissue injury. Excessive H_2_O_2_ production or insufficient antioxidant capacity can disrupt redox homeostasis, thereby promoting oxidative damage and apoptosis in thyrocytes and exacerbating pathological processes within thyroid tissue [7].

Alterations in thyroid status may disrupt redox homeostasis at the systemic level because thyroid hormones regulate energy metabolism, mitochondrial activity, oxygen consumption, lipid metabolism, and inflammatory responses. Consequently, both hypothyroidism and autoimmune inflammation may be accompanied by an imbalance between pro-oxidant processes and antioxidant defenses [8]. Redox disturbances associated with HT may extend beyond the thyroid gland, become detectable in the systemic circulation, and be associated with metabolic and vascular abnormalities. Therefore, the measurement of circulating markers of oxidative burden, antioxidant defense, and oxidative modification products in serum or plasma provides a biologically sound approach to assessing systemic redox status in these patients [9,10].

Catalase is a major first-line antioxidant enzyme that catalyzes the decomposition of H_2_O_2_ into water and molecular oxygen. Catalase activity reflects the H_2_O_2_-detoxifying capacity of the biological matrix being analyzed; however, it does not represent overall antioxidant defense and should therefore be interpreted in conjunction with other redox biomarkers [11,12].

Erythrocytes constitute a biologically informative matrix for assessing antioxidant responses because they continuously transport oxygen, undergo repeated cycles of oxygenation and deoxygenation, contain hemoglobin and heme iron that can participate in redox reactions, and possess a robust enzymatic system for H_2_O_2_ detoxification that includes catalase, glutathione peroxidases, and peroxiredoxins [13]. However, erythrocyte catalase should not be considered in isolation from other antioxidant systems. H_2_O_2_ detoxification in red blood cells also depends on glutathione peroxidase and associated reductive systems; therefore, changes in catalase activity may reflect broader remodeling of antioxidant defenses rather than an isolated enzymatic response [14].

Plasma catalase activity reflects catalase present in the extracellular blood compartment but may be affected by the release of intracellular catalase, including that resulting from erythrocyte contamination of plasma samples. This measurement therefore requires careful control for hemolysis and should not be interpreted as a tissue-specific biomarker. Plasma catalase activity is best evaluated in conjunction with circulating markers of inflammation, oxidative protein damage, and total antioxidant capacity [15]. Concurrent assessment of erythrocyte catalase activity and plasma catalase activity may help distinguish the cellular and extracellular components of antioxidant defense, which is particularly important in diseases in which systemic inflammation and hormonal and metabolic disturbances may occur simultaneously.

In addition to oxidative stress, increasing attention has been given to the role of carbonyl stress in the pathophysiology of chronic endocrine and immune-mediated inflammatory diseases. Carbonyl stress results from the accumulation of reactive dicarbonyl compounds and the subsequent formation of advanced glycation end products (AGEs) [16]. Methylglyoxal (MGO) is one of the most reactive α-dicarbonyl metabolites. It is generated primarily through the nonenzymatic degradation of glycolytic triose phosphates and can modify proteins, nucleic acids, and lipids, thereby contributing to dicarbonyl stress [17].

The biological significance of MGO extends beyond its capacity to generate AGEs. MGO is also involved in protein dysfunction, oxidative damage, inflammatory signaling, and vascular and metabolic complications, making it a potentially informative marker of systemic metabolic stress [18]. The principal pathway of MGO detoxification is the glyoxalase system, which comprises glyoxalase I and glyoxalase II and depends on reduced glutathione (GSH). Thus, MGO concentrations are determined not only by the rate of MGO formation but also by the efficiency of cellular detoxification mechanisms [19].

Erythrocytes are particularly informative for assessing MGO-associated carbonyl status because they exhibit high glycolytic activity, contain glutathione-dependent antioxidant systems, and possess a functional glyoxalase pathway that may be modulated by glucose and other metabolic factors [20]. Measurement of MGO in the erythrocyte compartment may therefore reflect not only the overall degree of carbonyl stress but also the balance among intracellular MGO formation, binding, redistribution, and detoxification.

Carbonyl stress is closely intertwined with oxidative stress. Dicarbonyl compounds and AGEs can increase the generation of reactive oxygen species, activate inflammatory signaling, and damage cellular proteins, whereas oxidative stress can, in turn, promote glycation and the formation of reactive carbonyl compounds [21]. From this perspective, the concurrent assessment of catalase and MGO is biologically justified: catalase activity reflects the H_2_O_2_-detoxifying component of antioxidant defense, whereas MGO concentration reflects the dicarbonyl component of metabolic injury.

This combined approach is particularly relevant to thyroid disease because thyroid functional status influences energy metabolism, glycolysis, lipid metabolism, and mitochondrial processes, whereas the autoimmune component of the disease may sustain chronic immune–inflammatory activation and redox-dependent mechanisms of tissue injury [22]. Most previous studies of Hashimoto thyroiditis and hypothyroidism have focused on individual markers of oxidative stress, lipid peroxidation, total antioxidant capacity, or the glutathione system. Data on the simultaneous measurement of catalase activity and MGO concentration in plasma and erythrocytes across different clinical groups of thyroid disease remain limited.

Separate analysis of the plasma and erythrocyte compartments is essential because these biological matrices are not interchangeable. Plasma reflects the systemic extracellular pool of metabolites, proteins, and inflammatory mediators, whereas erythrocytes reflect the cellular redox–carbonyl response within a hemoglobin-rich intracellular environment continuously exposed to oxygen [23]. Accordingly, concordant or divergent changes in catalase activity and MGO concentrations in plasma and erythrocytes may indicate different patterns of systemic metabolic adaptation in thyroid disease.

Despite growing evidence linking thyroid disorders to oxidative and glycoxidative stress, it remains unclear whether alterations in catalase activity and methylglyoxal (MGO) concentration are primarily associated with thyroid hormone deficiency, autoimmune inflammation, or structural thyroid pathology, or represent more general features of thyroid disease [24].

The clinical heterogeneity of autoimmune thyroiditis is particularly relevant in this context because autoimmune thyroid disease may occur with preserved thyroid function or progress from subclinical to overt hypothyroidism, reflecting different combinations of immune-mediated thyroid injury and functional impairment [25].

Accordingly, the present study included four clinically distinct thyroid phenotypes representing different combinations of functional, autoimmune, and structural abnormalities: hypothyroidism without autoimmune thyroiditis, autoimmune thyroiditis with hypothyroidism, euthyroid autoimmune thyroiditis, and nontoxic nodular goiter. The inclusion of nontoxic nodular goiter provided a structural thyroid-disease phenotype characterized by nodular changes in the absence of overt thyroid dysfunction and autoimmune thyroiditis, thereby complementing the functional and autoimmune phenotypes included in the study [26].

This phenotype-based design was therefore intended to help distinguish biochemical changes associated predominantly with thyroid functional impairment from those potentially related to autoimmune inflammation or structural thyroid pathology.

The biological rationale for this comparison is supported by the close relationship between thyroid function, cellular metabolism, and redox homeostasis. Thyroid hormones are major regulators of energy metabolism and influence nutrient utilization, thermogenesis, mitochondrial function, and systemic metabolic activity [27].

Autoimmune thyroiditis may additionally contribute to redox imbalance through persistent immune–inflammatory activation and oxidative injury, and alterations in oxidant–antioxidant balance have been described across different functional stages of autoimmune thyroid disease [28].

Catalase represents a specific component of antioxidant defense because it catalyzes the decomposition of hydrogen peroxide into water and molecular oxygen, thereby contributing directly to H_2_O_2_ detoxification [29].

MGO, in contrast, is a reactive dicarbonyl metabolite generated predominantly from glycolytic intermediates, while its major detoxification pathway depends on the glutathione-associated glyoxalase system [30].

Oxidative and carbonyl stress are biologically interconnected because increased MGO and MGO-derived advanced glycation end products can promote pro-oxidant signaling and reactive oxygen species generation, providing a mechanistic basis for evaluating catalase activity and MGO concentration together [31].

Finally, erythrocytes and plasma should be considered separately because the erythrocyte compartment has a specialized glycolytic and redox metabolism, and direct metabolomic comparisons demonstrate substantial differences between red blood cell and plasma metabolic profiles [32].

The central research question was whether catalase activity and MGO concentration differ according to thyroid functional status and the presence or absence of autoimmune thyroiditis and whether these patterns depend on the blood compartment examined. We hypothesized that catalase activity and MGO concentration would exhibit phenotype- and compartment-dependent patterns rather than a uniform response across all thyroid disorders. Accordingly, the aim of this study was to compare catalase activity and MGO concentrations in erythrocytes and plasma across functional, autoimmune, and structural thyroid phenotypes and to characterize the correlations among these variables within and between the erythrocyte and plasma compartments.

## 2. Materials and Methods

### 2.1. Study Design

This single-center, cross-sectional observational study was conducted at the Biochemistry Laboratory of the Department of Biomedicine, Karaganda Medical University, between 2022 and 2024.

Catalase activity and methylglyoxal concentration were assessed in two blood compartments—the erythrocyte fraction and plasma. This design was used to compare the cellular and extracellular components of redox–carbonyl status between control participants and patients with different functional, autoimmune, and structural phenotypes of thyroid disease.

The primary objective was to compare catalase activity and MGO concentration across five predefined clinical groups representing different combinations of thyroid functional, autoimmune, and structural characteristics.

### 2.2. Study Groups

A total of 164 participants were included in the study. Participants were assigned to five predefined groups according to thyroid functional status and the presence or absence of an autoimmune component:

Group 1—the control group (*n* = 24): participants with no clinically diagnosed thyroid disease, normal thyroid function test results, and no laboratory evidence of autoimmune thyroiditis.

Group 2—patients with hypothyroidism without autoimmune thyroiditis (Hypo-nonAIT; *n* = 31): patients with subclinical or overt hypothyroidism and no serological evidence of autoimmune thyroiditis.

Group 3—patients with autoimmune thyroiditis with hypothyroidism (AIT-Hypo; *n* = 30): patients with subclinical or overt hypothyroidism accompanied by seropositivity for anti-thyroid peroxidase antibodies (anti-TPO) and/or anti-thyroglobulin antibodies (anti-TG).

Group 4—patients with nontoxic nodular goiter with euthyroidism (NNG, euthyroid; *n* = 42): patients with thyroid nodules identified by ultrasonography, no evidence of autoimmune thyroid involvement, normal thyroid function, and no clinical or laboratory evidence of thyroid dysfunction. The laboratory criteria were TSH 0.40–4.00 mIU/L, fT4 10–22 pmol/L, anti-TPO < 35 IU/mL, and anti-TG < 115 IU/mL.

Group 5—patients with euthyroid autoimmune thyroiditis (Euth-AIT; *n* = 37): patients with euthyroidism accompanied by seropositivity for anti-TPO and/or anti-TG.

Participants were assigned to the study groups according to their clinical diagnosis, thyroid functional status, and the presence or absence of an autoimmune component. Sex- and age-stratified analyses were not prespecified because the study was designed around the five clinical phenotypes and further stratification would have produced small subgroups. All participants were within the predefined age range of 18–59 years.

### 2.3. Operational Definitions of Clinical Phenotypes

Euthyroidism was defined as a thyroid-stimulating hormone (TSH) concentration of 0.40–4.00 mIU/L in combination with a free thyroxine (fT4) concentration of 10–22 pmol/L.

Subclinical hypothyroidism was defined as TSH > 4.00 mIU/L with an fT4 concentration within the reference range.

Overt hypothyroidism was defined as TSH > 4.00 mIU/L accompanied by an fT4 concentration < 10 pmol/L.

Autoimmune seropositivity was defined as an anti-TPO concentration ≥ 35 IU/mL and/or an anti-TG concentration ≥ 115 IU/mL, as determined by quantitative enzyme-linked immunosorbent assay.

Autoimmune thyroiditis was diagnosed by an endocrinologist based on an anti-TPO concentration ≥ 35 IU/mL and/or an anti-TG concentration ≥ 115 IU/mL. Ultrasonographic features of chronic autoimmune thyroid involvement were considered supportive diagnostic evidence. The same classification algorithm was applied to all participants.

Levothyroxine treatment status was recorded at enrollment. Among patients with hypothyroidism without autoimmune thyroiditis, 22 of 31 (71.0%) were receiving levothyroxine, whereas 9 (29.0%) were not receiving levothyroxine at the time of enrollment. Among patients with autoimmune thyroiditis and hypothyroidism, 24 of 30 (80.0%) were receiving levothyroxine and 6 (20.0%) were not. All participants assigned to the two hypothyroid groups met the predefined biochemical criteria for subclinical or overt hypothyroidism at enrollment, irrespective of treatment status. Thus, patients receiving levothyroxine had persistent biochemical hypothyroidism despite ongoing replacement therapy at the time of assessment. In treated patients, venous blood was collected in the morning before the next scheduled levothyroxine dose. Levothyroxine dose and treatment duration were recorded at enrollment.

### 2.4. Inclusion and Exclusion Criteria

The inclusion criteria were as follows: age 18–59 years; availability of TSH, fT4, anti-TPO, and anti-TG results and an endocrinologist’s clinical assessment sufficient to assign the participant to one of the study groups; the ability to classify the participant according to thyroid functional and autoimmune status; availability of blood samples suitable for measuring catalase activity and methylglyoxal concentration in plasma and the erythrocyte fraction; and written informed consent to participate in the study.

The exclusion criteria were as follows: age < 18 or >59 years; pregnancy or lactation; acute infectious or inflammatory disease within 4 weeks before blood collection; exacerbation of a chronic medical condition; concomitant endocrine disorders, including thyrotoxicosis, subacute thyroiditis, type 1 or type 2 diabetes mellitus, and other clinically significant endocrine disorders; severe cardiovascular, hepatic, or renal failure; active malignancy; clinically significant hematologic disorders; and the use of medications or dietary supplements with substantial antioxidant activity within 4 weeks before blood collection.

Medication and dietary supplement use during the preceding 4 weeks was recorded for all participants using a standardized questionnaire. Participants taking antioxidant preparations, including vitamins C and E and N-acetyl-L-cysteine (NAC), as well as medications capable of affecting antioxidant enzyme activity or the concentrations of reactive carbonyl compounds, including methylglyoxal, were excluded.

Biological samples were excluded from laboratory analysis in cases of marked hemolysis, lipemia, icterus, repeated freeze–thaw cycles, or noncompliance with preanalytical storage and transportation requirements.

### 2.5. Ethical Considerations

The study was conducted in accordance with the principles of the Declaration of Helsinki and local requirements governing biomedical research involving human participants. The study protocol was approved by the Ethics Committee of Karaganda Medical University (Protocol No. 1, dated 18 September 2022).

All participants were informed of the study objectives and provided written informed consent for participation and for the use of deidentified biological specimens.

### 2.6. Assessment of Thyroid Profile

Thyroid functional status was assessed based on clinical examination findings and laboratory thyroid profile measurements, including TSH, fT4, and, when clinically indicated, free triiodothyronine (fT3).

Autoimmune status was assessed based on anti-TPO and anti-TG concentrations. Thyroid hormones and thyroid autoantibodies were measured using quantitative enzyme-linked immunosorbent assays according to the diagnostic kit manufacturers’ instructions.

TSH values were expressed in mIU/L, fT4 in pmol/L, and anti-TPO and anti-TG in IU/mL. Thyroid functional and autoimmune status was classified using the laboratory reference intervals and diagnostic cutoff values specified above.

### 2.7. Blood Collection and Sample Preparation

Venous blood was collected in the morning after an overnight fast of 8–12 h into evacuated tubes containing lithium heparin. Blood samples were processed within 60 min of collection and centrifuged at 2000× *g* for 10 min at 4 °C. Plasma was immediately separated from the cellular fraction and aliquoted within the same 60-min preanalytical interval. Samples intended for analysis within 48 h were stored at 2–8 °C; when longer storage was required, plasma aliquots were frozen at −80 °C until analysis. The maximum storage duration at 2–8 °C was 48 h. Repeated freeze–thaw cycles were not permitted.

After plasma separation, packed erythrocytes were washed three times with chilled 0.9% NaCl solution at 4 °C using a packed-cell-to-saline ratio of 1:3 (*v*/*v*), followed by resuspension and centrifugation at 3000× *g* for 10 min at 4 °C.

To prepare the hemolysate, one volume of washed packed erythrocytes was mixed with nine volumes of chilled distilled water at 4 °C to induce hypotonic hemolysis. The resulting hemolysate was used to measure erythrocyte catalase activity and erythrocyte methylglyoxal concentration.

Samples showing marked hemolysis, lipemia, icterus, or evidence of improper storage were excluded from the analysis. The interval between blood collection and sample processing and the absence of repeated freeze–thaw cycles were monitored for all samples.

### 2.8. Determination of Catalase Activity in Plasma and Erythrocytes

Catalase activity (EC 1.11.1.6) in plasma and erythrocyte hemolysate was measured spectrophotometrically using a modified ammonium molybdate method based on the procedures described by Koroliuk et al. (1988) and Góth (1991) [33,34]. The method is based on enzymatic decomposition of hydrogen peroxide (H_2_O_2_), followed by spectrophotometric quantification of the residual H_2_O_2_ through formation of a colored complex with ammonium molybdate.

For the assay, 1.0 mL of freshly prepared 30 mM H_2_O_2_ solution was mixed with 0.1 mL of plasma or erythrocyte hemolysate and incubated for 60 s at 25 °C. The reaction was terminated by addition of 1.0 mL of 4% ammonium molybdate solution. A corresponding control reaction was prepared for each biological sample by adding ammonium molybdate before the biological material to prevent enzymatic decomposition of H_2_O_2_. A reagent blank contained all assay components except the biological sample.

Before spectrophotometric measurement, reaction mixtures were diluted 20-fold with distilled water. Absorbance was measured at 410 nm in a 1 cm optical path-length cuvette using a PD-303UV digital UV–Vis spectrophotometer (APEL Co., Ltd., Kawaguchi, Saitama, Japan). Plasma samples were initially analyzed without dilution and were diluted further only when necessary to bring absorbance within the validated analytical range.

Erythrocyte catalase activity was determined in the 1:10 erythrocyte hemolysate prepared as described in Section 2.7 and was normalized to hemoglobin concentration measured in a separate aliquot of the same hemolysate using the cyanmethemoglobin method. Plasma catalase activity was expressed in mkat/L, whereas erythrocyte catalase activity was expressed in U/g Hb.

All biological samples were analyzed in duplicate, and samples were reanalyzed when the relative difference between duplicate measurements exceeded 10%. The within-run and between-run coefficients of variation were 4.8% and 7.2%, respectively. The validated working range of the plasma assay was 0.01–20.0 U/mL, corresponding to 0.000167–0.333 mkat/L. Detailed calibration procedures, calculation equations, dilution-factor corrections, unit conversions, hemoglobin normalization, and validation procedures are provided in Supplementary Methods S1.

### 2.9. Determination of Methylglyoxal Concentrations in Plasma and Erythrocytes

Methylglyoxal concentrations in plasma and erythrocyte hemolysate were measured spectrophotometrically after derivatization with N-acetyl-L-cysteine (NAC) using a substantially modified version of the method described by Wild et al. (2012), adapted and validated for MGO determination in the micromolar range [35]. The method is based on formation of an MGO–NAC derivative with an absorption maximum at 288 nm.

Plasma and erythrocyte hemolysate samples were deproteinized with 0.6 M perchloric acid at a 1:1 (*v*/*v*) ratio, incubated on ice for 10 min, and centrifuged at 10,000× *g* for 10 min at 4 °C. The resulting supernatant was neutralized to pH 7.0 with 2 M K_2_CO_3_, incubated on ice for a further 10 min, and centrifuged again under the same conditions. The clear supernatant was used for MGO determination.

For the derivatization reaction, 930 µL of 100 mM phosphate buffer (pH 7.0), 50 µL of the prepared biological sample, and 20 µL of freshly prepared 500 mM NAC solution were combined to obtain a final reaction volume of 1.0 mL. The mixture was incubated for 10 min at 25 °C, and absorbance was measured at 288 nm in a 1 cm quartz cuvette using a PD-303UV digital UV–Vis spectrophotometer (APEL, Japan). Matrix controls without NAC and reagent blanks without biological material were analyzed in parallel to correct for background absorbance.

Final plasma MGO concentrations were expressed in µmol/L. Erythrocyte MGO concentrations were normalized to hemoglobin concentration measured in the corresponding original hemolysate and expressed in nmol/g Hb.

All biological samples were analyzed in duplicate, and samples were reanalyzed when the relative difference between duplicate measurements exceeded 10%. For plasma, the limit of detection and limit of quantification were 0.002 and 0.005 µmol/L, respectively, with within-run and between-run coefficients of variation of 5.2% and 7.8%. For erythrocyte hemolysate, the corresponding values were 0.003 and 0.008 µmol/L, with coefficients of variation of 6.1% and 8.9%. Detailed calibration equations, dilution-factor calculations, hemoglobin-normalization equations, spike-and-recovery procedures, assessment of matrix effects, analytical linearity, dilutional parallelism, and analytical sensitivity are provided in Supplementary Methods S2.

### 2.10. Quality Control of Laboratory Measurements

All samples were coded and analyzed with the analyst blinded to group assignment. Samples from the five groups were distributed evenly across analytical runs. Each run included a reagent blank, calibrators, and low- and high-level control materials. Biological samples were analyzed in duplicate, and the analysis was repeated when the difference between duplicate measurements exceeded 10%. All samples were processed in coded, deidentified form.

### 2.11. Statistical Analysis

No a priori sample size calculation was performed. The sample size was determined by the number of eligible participants who met the predefined inclusion and exclusion criteria and were enrolled during the predefined study period; therefore, the study was not based on a prespecified statistical power target.

Statistical analyses were performed using GraphPad Prism version 8.0.1. The distribution of each variable within each group was evaluated before selecting the method for between-group comparisons. Normality was assessed using the D’Agostino–Pearson and Shapiro–Wilk tests. Age was compared across the five study groups using one-way analysis of variance (ANOVA), and sex distribution was compared using Pearson’s chi-square test. Age, sex, and BMI were not included as covariates in the primary phenotype-based analyses; therefore, possible residual confounding by these variables was considered when interpreting the results. Smoking status and habitual iodine intake were not systematically assessed and could not be included in adjusted analyses.

Because most study variables were not normally distributed, quantitative data were presented as median [Q1–Q3]. Minimum and maximum values (Min–Max) were also reported in the tables to characterize the full range of individual variability.

The actual number of observations (n) was reported for each variable because individual samples could be excluded because of preanalytical defects or insufficient sample volume.

The five independent groups were compared using the nonparametric Kruskal–Wallis test. When the overall test indicated statistically significant between-group differences, multiple comparisons were performed using Dunn’s post hoc test. Adjusted *p* values were used for all multiple comparisons. For each Kruskal–Wallis analysis, effect size was estimated using epsilon-squared (ε^2^), calculated as ε^2^ = (H − k + 1)/(n − k), where H is the Kruskal–Wallis statistic, k is the number of groups, and n is the number of observations included in the analysis.

Associations between the study variables were evaluated using Spearman rank correlation analysis. Four variable pairs were predefined: erythrocyte catalase activity and erythrocyte methylglyoxal concentration; plasma catalase activity and plasma methylglyoxal concentration; erythrocyte catalase activity and plasma catalase activity; and erythrocyte methylglyoxal concentration and plasma methylglyoxal concentration. Correlations were calculated separately for the control group, each of the four patient phenotypes, the pooled group of patients with thyroid disease, and the combined analytic sample comprising all study participants. The phenotype-specific analyses were performed to evaluate whether the correlation patterns observed in the pooled patient group were consistent across the individual clinical phenotypes. The pooled patient analysis was used as a secondary analysis to evaluate whether common associations between the investigated variables were detectable across thyroid disease as a whole rather than within individual clinical phenotypes. Because the patient group comprised heterogeneous thyroid phenotypes, pooled correlations were interpreted as overall patient-level associations and not as phenotype-specific effects. To control the family-wise error rate arising from the four predefined correlation tests within each analyzed sample, *p* values were adjusted using the Holm method. Holm-adjusted *p* values were reported together with Spearman’s rank correlation coefficient (r_s_) and its 95% confidence interval.

## 3. Results

### 3.1. Baseline Demographic and Clinical Characteristics

The study included 164 participants distributed among five predefined clinical groups: hypothyroidism without autoimmune thyroiditis (*n* = 31), autoimmune thyroiditis with hypothyroidism (*n* = 30), nontoxic nodular goiter with euthyroidism (*n* = 42), euthyroid autoimmune thyroiditis (*n* = 37), and controls (*n* = 24). Baseline demographic, clinical, laboratory, and treatment characteristics of the study groups are summarized in Table 1. Age did not differ significantly among the five groups (one-way ANOVA, F(4159) = 1.01, *p* = 0.403), and sex distribution was also comparable across groups (Pearson’s χ^2^(4) = 4.06, *p* = 0.399). Women predominated in all five groups (Table 1).

Median BMI ranged from 25.8 kg/m^2^ in controls to 29.3 kg/m^2^ in patients with hypothyroidism without autoimmune thyroiditis. As expected from the predefined clinical phenotypes, TSH and fT4 concentrations reflected thyroid functional status, whereas anti-TPO and anti-TG concentrations were markedly higher in the two autoimmune thyroiditis groups. Subclinical hypothyroidism predominated over overt hypothyroidism in both hypothyroid groups. Levothyroxine was used by 71.0% of patients with hypothyroidism without autoimmune thyroiditis and by 80.0% of patients with autoimmune thyroiditis and hypothyroidism; the median doses were 1.00 and 0.91 µg/kg/day, respectively, and the corresponding median treatment durations were 39.5 and 46.5 months. Median eGFR values were above 90 mL/min/1.73 m^2^ in all study groups.

### 3.2. Erythrocyte Catalase Activity

Erythrocyte catalase activity differed significantly among the study groups (H(4) = 82.98, *p* < 0.0001, ε^2^ = 0.497) (Table 2).

Compared with the control group, median erythrocyte catalase activity was 8.45-fold higher in patients with hypothyroidism without autoimmune thyroiditis (*p* = 0.0025), 11.25-fold higher in patients with autoimmune thyroiditis and hypothyroidism (*p* < 0.0001), 10.94-fold higher in patients with nontoxic nodular goiter (*p* < 0.0001), and 11.37-fold higher in patients with euthyroid autoimmune thyroiditis (*p* < 0.0001).

Among the patient groups, the lowest median erythrocyte catalase activity was observed in patients with hypothyroidism without autoimmune thyroiditis. Relative to this group, the median value was 33.2% higher in patients with autoimmune thyroiditis and hypothyroidism (*p* = 0.0009), 29.5% higher in patients with nontoxic nodular goiter (*p* = 0.0028), and 34.7% higher in patients with euthyroid autoimmune thyroiditis (*p* = 0.0005).

No statistically significant differences were observed among the AIT + hypothyroidism, NNG euthyroid, and euthyroid AIT groups.

Figure 1 shows the distribution of erythrocyte catalase activity across the study groups. Values in the control group were lower than those observed in all patient groups, whereas the hypothyroidism without autoimmune thyroiditis group had a lower median than the other three patient groups (Figure 1).

Overall, erythrocyte catalase activity was significantly higher in all four patient groups than in controls. However, the increase was less pronounced in patients with hypothyroidism without autoimmune thyroiditis than in the other three patient groups.

### 3.3. Plasma Catalase Activity

Plasma catalase activity differed significantly among the study groups (H(4) = 19.77, *p* = 0.0006, ε^2^ = 0.099) (Table 3).

Post hoc pairwise comparisons showed significantly higher plasma catalase activity in the Hypothyroidism without AIT group (*p* = 0.0262) and the NNG euthyroid group (*p* = 0.0002) compared with controls. Plasma catalase activity was numerically higher in the AIT + hypothyroidism and Euthyroid AIT groups than in Controls, but these differences were not statistically significant (*p* = 0.1473 and *p* = 0.1544, respectively). No statistically significant differences were observed among the patient groups. Figure 2 shows the distribution of plasma catalase activity across the study groups. Median values were numerically higher in all patient groups than in controls, although the distributions of the patient groups overlapped substantially (Figure 2).

Overall, plasma catalase activity was significantly higher than in Controls in the Hypothyroidism without AIT and NNG euthyroid groups. The two AIT groups showed numerically higher median values, but the differences from controls were not statistically significant, and no significant differences were observed among the patient groups.

### 3.4. Erythrocyte Methylglyoxal Concentration

Erythrocyte MGO concentration differed significantly among the study groups (H(4) = 12.66, *p* = 0.0131, ε^2^ = 0.054) (Table 4).

Post hoc pairwise comparisons showed significantly lower erythrocyte MGO concentrations in the Hypothyroidism without AIT group (*p* = 0.0292) and the NNG euthyroid group (*p* = 0.0058) compared with Controls. The difference between Controls and the AIT + hypothyroidism group did not reach statistical significance (*p* = 0.0517), and the Euthyroid AIT group did not differ significantly from Controls (*p* = 0.3611). No statistically significant differences were identified among the patient groups. Figure 3 shows the distribution of erythrocyte MGO concentrations across the study groups. Median values were numerically lower in all patient groups than in Controls, although the distributions overlapped substantially (Figure 3).

Overall, erythrocyte MGO concentration was significantly lower than in Controls in the Hypothyroidism without AIT and NNG euthyroid groups. The difference for AIT + hypothyroidism did not reach statistical significance (*p* = 0.0517), whereas Euthyroid AIT did not differ significantly from controls. No significant differences were observed among the patient groups.

### 3.5. Plasma Methylglyoxal Concentration

Plasma MGO concentration did not differ significantly among the study groups (H(4) = 4.419, *p* = 0.3522, ε^2^ = 0.003) (Table 5).

Plasma MGO concentration did not differ significantly among the five study groups (H(4) = 4.419, *p* = 0.3522). Median values ranged from 0.2250 µmol/L in the NNG group to 0.2560 µmol/L in the Euth-AIT group, with substantial overlap of the distributions across groups (Figure 4).

Overall, plasma methylglyoxal concentration did not differ significantly among the study groups. The descriptively higher median values observed in the two autoimmune thyroiditis groups were not supported by statistically significant between-group differences.

### 3.6. Correlations Between Catalase Activity and Methylglyoxal Concentrations

Correlations between catalase activity and methylglyoxal concentrations were assessed using Spearman rank correlation analysis. The primary correlation analysis was performed separately in the control group, the pooled group of patients with thyroid disease, and the combined analytic sample (Table 6).

To further examine phenotype-specific correlation patterns and facilitate visual comparison, Spearman correlation coefficients were additionally calculated separately for each of the four patient groups and are presented together with the three primary analytic samples in Figure 5.

As illustrated in Figure 5, the phenotype-specific correlation patterns were heterogeneous across the four patient groups. No within-compartment association between catalase activity and MGO concentration remained statistically significant after Holm correction in any individual patient phenotype. The strongest phenotype-specific association was observed in the NNG group, in which erythrocyte and plasma MGO concentrations were strongly positively correlated (r_s_ = 0.723; 95% CI, 0.537 to 0.842; Holm-adjusted *p* < 0.0001). No other phenotype-specific correlation remained statistically significant after Holm correction.

In the control group, erythrocyte catalase activity showed a weak positive correlation with erythrocyte MGO concentration, but the association was not statistically significant (r_s_ = 0.145; 95% CI, −0.275 to 0.518; Holm-adjusted *p* = 1.000). Erythrocyte and plasma catalase activities were also not significantly correlated (r_s_ = 0.078; 95% CI, −0.336 to 0.466; Holm-adjusted *p* = 1.000). Plasma catalase activity showed a moderate negative correlation with plasma MGO concentration (r_s_ = −0.479; 95% CI, −0.739 to −0.094); however, this association did not remain statistically significant after Holm correction (*p* = 0.0537). In contrast, erythrocyte and plasma MGO concentrations showed a very strong positive correlation (r_s_ = 0.950; 95% CI, 0.887 to 0.979; Holm-adjusted *p* < 0.0001).

In patients with thyroid disease, erythrocyte catalase activity showed a weak negative correlation with erythrocyte MGO concentration, which was not statistically significant (r_s_ = −0.113; 95% CI, −0.274 to 0.054; Holm-adjusted *p* = 0.3689). Plasma catalase activity showed a weak positive correlation with plasma MGO concentration, which was also not statistically significant (r_s_ = 0.139; 95% CI, −0.028 to 0.298; Holm-adjusted *p* = 0.3068). There was virtually no correlation between erythrocyte and plasma catalase activities (r_s_ = −0.005; 95% CI, −0.171 to 0.161; Holm-adjusted *p* = 0.9519). In contrast, erythrocyte and plasma MGO concentrations showed a moderate positive correlation (r_s_ = 0.392; 95% CI, 0.241 to 0.523; Holm-adjusted *p* < 0.0001).

In the combined analytic sample, erythrocyte catalase activity was moderately negatively correlated with erythrocyte MGO concentration (r_s_ = −0.420; 95% CI, −0.538 to −0.285; Holm-adjusted *p* < 0.0001). Erythrocyte and plasma catalase activities showed a moderate positive correlation (r_s_ = 0.369; 95% CI, 0.228 to 0.494; Holm-adjusted *p* < 0.0001). A weak positive correlation was observed between plasma catalase activity and plasma MGO concentration (r_s_ = 0.172; 95% CI, 0.020 to 0.317; Holm-adjusted *p* = 0.0274). Erythrocyte and plasma MGO concentrations were also weakly positively correlated (r_s_ = 0.246; 95% CI, 0.097 to 0.385; Holm-adjusted *p* = 0.0029).

Although all four correlations reached statistical significance in the combined analytic sample, the three catalase-related associations were not reproduced within both the control group and the pooled patient group. In particular, the direction of the association between plasma catalase activity and plasma MGO concentration differed between controls and patients. Therefore, the catalase-related correlations detected in the combined sample should be interpreted cautiously, because they may be influenced substantially by between-group separation rather than reflecting uniform individual-level relationships throughout the study population.

In contrast, the positive association between erythrocyte and plasma MGO concentrations was observed in all three primary analyses. Although its magnitude ranged from very strong in controls to moderate in pooled patients and weak in the combined analytic sample, it remained statistically significant after correction for multiple testing in all three primary analyses. The phenotype-specific analysis, however, showed heterogeneity across the individual patient groups: this association remained statistically significant after Holm correction only in the NNG group.

Overall, the correlation analysis did not identify a statistically significant within-compartment association between catalase activity and MGO concentration in the pooled group of patients with thyroid disease. The most reproducible finding was the positive association between erythrocyte and plasma MGO concentrations. Catalase-related correlations were significant only in the combined analytic sample and therefore appeared to depend on the composition of the analyzed population.

## 4. Discussion

The present study identified differences in catalase activity and methylglyoxal concentration between the control group and patients with thyroid disease, with the patterns of change depending on the biological compartment examined. Erythrocyte catalase activity was higher in all patient groups than in Controls, whereas plasma catalase activity was significantly higher than in controls only in the Hypothyroidism without AIT and NNG euthyroid groups; the two AIT groups showed numerically higher plasma catalase activity without statistically significant differences from controls. Erythrocyte catalase activity in patients with hypothyroidism without autoimmune thyroiditis was lower than that in patients with autoimmune thyroiditis with hypothyroidism, nontoxic nodular goiter, and euthyroid autoimmune thyroiditis. No differences in plasma catalase activity were observed among the patient groups. Erythrocyte MGO concentration was significantly lower than in Controls in the Hypothyroidism without AIT and NNG euthyroid groups; the difference for AIT + hypothyroidism did not reach statistical significance, whereas Euthyroid AIT did not differ significantly from controls. Plasma MGO concentration showed no statistically significant between-group differences. Thus, catalase activity and methylglyoxal concentration showed distinct between-group profiles in plasma and erythrocytes.

H_2_O_2_ production is required for thyroid peroxidase-dependent iodination of thyroglobulin and thyroid hormone synthesis; however, excessive generation of reactive oxygen species or insufficient detoxification capacity may contribute to thyrocyte injury and autoimmune inflammation. Autoimmune thyroiditis is not characterized by a uniform redox profile, because the magnitude of redox alterations depends on thyroid functional status, metabolic context, nutritional status, body composition, and immune activity. Therefore, the increased catalase activity observed in the present study is more consistent with a systemic adaptive response to thyroid disease than with direct evidence of either exclusively increased oxidative injury or fully effective antioxidant protection [36].

Increased catalase activity in blood may reflect compensatory enhancement of the H_2_O_2_-detoxifying component of the antioxidant system. However, the activity of a single enzyme does not characterize overall redox balance and cannot independently determine whether the antioxidant response is sufficient to limit cellular injury. In erythrocytes, catalase interacts functionally with glutathione peroxidase and peroxiredoxin 2; reduced activity of one enzyme may be accompanied by redistribution of the antioxidant burden among other components of the antioxidant network. In addition, erythrocyte catalase activity may also be modified by oxidative and nitrosative post-translational changes, including S-nitrosylation and glutathionylation [37,38].

Differences in erythrocyte catalase activity among the patient groups are of particular interest. The lower enzyme activity observed in patients with hypothyroidism without autoimmune thyroiditis than in the other three patient groups may reflect differences in the combined hormonal, metabolic, and inflammatory effects on erythrocytes. Thyroid hormones exert systemic effects on carbohydrate and lipid metabolism, oxygen consumption, and the generation of reactive oxygen species in tissues. Mature erythrocytes lack mitochondria; therefore, the effects of thyroid status on their antioxidant enzymes are likely mediated indirectly through changes in the systemic metabolic and inflammatory environment, the composition of the circulating erythrocyte population, and oxygenation conditions. At the same time, recent metabolomic studies of autoimmune thyroiditis have demonstrated alterations in carbohydrate, lipid, and amino acid metabolism even in the euthyroid state. However, in the present study, high erythrocyte catalase activity was observed not only in the autoimmune thyroiditis groups but also in patients with nontoxic nodular goiter, who had preserved thyroid function and no laboratory evidence of an autoimmune process. Therefore, the observed pattern cannot be attributed exclusively to autoimmunity or thyroid hormone deficiency and more likely reflects the combined effects of several systemic factors [39].

In contrast, plasma catalase activity was significantly higher than in Controls in the Hypothyroidism without AIT and NNG euthyroid groups, whereas the numerically higher values in the AIT + hypothyroidism and Euthyroid AIT groups did not reach statistical significance. No statistically significant differences were observed among the four patient groups. Thus, the present findings do not support a uniform increase in plasma catalase activity across all thyroid disease phenotypes. Nevertheless, plasma catalase should not be considered a tissue-specific or diagnostic biomarker. Catalase is predominantly an intracellular enzyme and is particularly abundant in erythrocytes; therefore, its measured activity in plasma may be sensitive to the release of intracellular components. Exclusion of visibly hemolyzed samples reduces this risk but does not eliminate the potential influence of occult hemolysis, because cell-free hemoglobin and other erythrocyte-derived components may be present in plasma before visible discoloration occurs [40].

The lower erythrocyte MGO concentrations observed in the patient groups, particularly the statistically significant reductions in the Hypothyroidism without AIT and NNG euthyroid groups, merit closer examination. Methylglyoxal is a highly reactive, short-lived α-dicarbonyl metabolite generated primarily through the nonenzymatic degradation of triose phosphates. Its concentration is determined not only by its rate of formation but also by glyoxalase system activity, the availability of reduced glutathione, binding to proteins and nucleic acids, and the formation of stable MGO-derived products. Because MGO was measured after deproteinization of the hemolysate in the present study, the resulting value primarily represents the extractable low-molecular-weight pool and does not directly reflect the amount of MGO already bound to hemoglobin or other proteins. Therefore, a decrease in measured erythrocyte MGO concentration is not equivalent to demonstrated reduction in overall carbonyl damage [41,42].

The observed reductions in erythrocyte MGO concentration may have several biologically plausible explanations, including reduced MGO formation as a consequence of altered glycolytic flux, enhanced glutathione-dependent detoxification through the glyoxalase system, or increased binding of MGO to cellular proteins with the formation of stable adducts. Because glyoxalase I and glyoxalase II activities change during erythrocyte maturation and aging, the measured MGO concentration may also be influenced by the age composition of the circulating erythrocyte population. These mechanisms were not directly evaluated in the present study and should therefore be regarded only as testable hypotheses [43,44].

No statistically significant differences in erythrocyte MGO concentration were detected among the patient groups. However, comparisons with Controls showed significant reductions only in the Hypothyroidism without AIT and NNG euthyroid groups; the difference for AIT + hypothyroidism did not reach statistical significance, whereas Euthyroid AIT did not differ significantly from controls. Therefore, the present data do not support a uniform decrease in erythrocyte MGO concentration across all thyroid disease phenotypes. Recent metabolomic studies have also shown that autoimmune thyroiditis is accompanied by multiple alterations in carbohydrate, lipid, amino acid, and energy metabolism. Consequently, a change in a single low-molecular-weight metabolite may depend on the broader metabolic context and may not necessarily parallel the distributions of TSH, fT4, or thyroid autoantibodies [45].

In contrast to the between-group differences observed for erythrocyte MGO, plasma MGO concentration did not distinguish among the functional and autoimmune phenotypes of thyroid disease. This discrepancy emphasizes that the cellular and circulating MGO pools reflect different metabolic processes. Free plasma MGO concentration represents the dynamic balance among its release from tissues and cells, glyoxalase-dependent detoxification, protein binding, and subsequent clearance. Therefore, even in the presence of local or intracellular disturbances in dicarbonyl metabolism, plasma MGO concentration may remain relatively stable because of compensatory redistribution and removal of the metabolite. The absence of statistically significant phenotypic differentiation also indicates that, in the present between-group analysis, autoimmune thyroiditis was not associated with statistically significant differences in plasma methylglyoxal concentration.

Plasma methylglyoxal concentration is likely influenced simultaneously by thyroid function, replacement therapy, glucose and lipid metabolism, glyoxalase system activity, reduced glutathione availability, and individual differences in clearance. Such multifactorial regulation may explain the pronounced within-group variability and overlap of distributions among the clinical phenotypes. Recent studies of the metabolic profile of autoimmune thyroiditis have also confirmed the heterogeneity of the systemic metabolic response and demonstrated that individual circulating variables do not necessarily form a specific profile directly corresponding to the presence of thyroid autoantibodies or thyroid functional status [46].

The absence of clear phenotypic specificity in plasma MGO concentration is consistent with the heterogeneity of published findings on the systemic glycoxidative profile of autoimmune thyroiditis. Higher AGE levels and lower sRAGE levels have been reported in euthyroid patients who were not receiving levothyroxine than in control participants. In contrast, lower AGE levels and higher sRAGE levels have been reported in patients with autoimmune thyroiditis receiving levothyroxine replacement therapy. These differences indicate that the glycoxidative profile may depend on treatment, the metabolic characteristics of the study population, thyroid functional status, and the analytical variable being measured [47,48].

The expanded correlation analysis did not provide evidence of a statistically significant monotonic association between catalase activity and MGO concentration within the same biological compartment in the pooled group of patients with thyroid disease. Erythrocyte catalase activity showed a weak negative association with erythrocyte MGO concentration, whereas plasma catalase activity showed a weak positive association with plasma MGO concentration. Neither relationship remained statistically significant after correction for multiple testing.

Erythrocyte and plasma catalase activities were also essentially uncorrelated in patients. Thus, interindividual variation in erythrocyte catalase activity was not accompanied by corresponding rank-order variation in plasma catalase activity. This finding supports separate interpretation of catalase activity in the cellular and extracellular blood compartments.

In the control group, plasma catalase activity showed a moderate negative association with plasma MGO concentration, but this relationship narrowly failed to remain statistically significant after Holm correction. In patients, the corresponding association was weakly positive and not statistically significant. In the combined analytic sample, plasma catalase activity showed a weak positive correlation with plasma MGO concentration that remained statistically significant after correction for multiple testing. The differences in both direction and magnitude across the three analyses do not support a uniform relationship between plasma catalase activity and plasma MGO concentration. The statistically significant combined-sample association may instead be influenced by between-group differences in the distributions of these variables.

The absence of statistically significant catalase–MGO associations within the pooled patient group does not exclude biological interactions between antioxidant and carbonyl pathways. Free MGO concentration is determined by its rate of formation, glyoxalase-system activity, the availability of reduced glutathione, protein binding, the formation of MGO-derived adducts, redistribution, and clearance, whereas catalase activity reflects only one component of enzymatic H_2_O_2_ detoxification. Consequently, the relationship between these processes may be indirect, nonlinear, and modified by metabolic, inflammatory, and treatment-related factors [49].

In contrast to the catalase-related correlations, erythrocyte and plasma MGO concentrations showed a reproducible positive association. This relationship was very strong in the control group, moderate in patients with thyroid disease, and weak in the combined analytic sample. Nevertheless, it remained statistically significant after correction for multiple testing in all three analyses. The consistent direction of these correlations supports partial concordance between the cellular and extracellular MGO pools, although the considerable difference in correlation magnitude indicates heterogeneity in their joint distribution across the analyzed populations.

The positive correlation between erythrocyte and plasma MGO concentrations does not imply that these measurements are interchangeable. Their between-group profiles differed: erythrocyte MGO concentration was significantly lower than in Controls in the Hypothyroidism without AIT and NNG euthyroid groups, whereas plasma MGO concentration did not differ significantly among the study groups. Thus, concordance in individual rank order may coexist with distinct group-level distributions, supporting separate evaluation of the cellular and extracellular MGO pools.

All three catalase-related correlations reached statistical significance in the combined analytic sample: erythrocyte catalase activity was moderately inversely associated with erythrocyte MGO concentration, erythrocyte and plasma catalase activities were moderately positively associated, and plasma catalase activity was weakly positively associated with plasma MGO concentration. However, none of these relationships was reproduced simultaneously within the control group and the pooled patient group. Moreover, the direction of the plasma catalase–plasma MGO relationship differed between controls and patients. These combined-sample associations should therefore be interpreted cautiously because they are likely influenced substantially by between-group separation rather than representing uniform individual-level relationships throughout the study population.

Overall, the findings demonstrate different group-level patterns across the erythrocyte and plasma compartments. Erythrocyte catalase activity was higher in all patient groups, whereas significant increases in plasma catalase activity were confined to the Hypothyroidism without AIT and NNG euthyroid groups. Erythrocyte MGO concentration was significantly lower than in Controls in these same two groups, whereas plasma MGO concentration did not differ significantly among the study groups. The correlation analysis showed no statistically significant within-compartment association between catalase activity and MGO concentration in the pooled group of patients with thyroid disease, whereas erythrocyte and plasma MGO concentrations were positively correlated in the control group, in patients, and in the combined analytic sample. Although all three catalase-related correlations were statistically significant in the combined sample, their absence or inconsistency within the separately analyzed control and patient groups suggests a substantial influence of between-group separation. Further studies should simultaneously assess the glyoxalase system, glutathione status, glycation products, markers of oxidative damage, and relevant clinical confounding factors to clarify the mechanisms underlying these compartment-dependent patterns.

## 5. Conclusions

In the present cross-sectional study, we evaluated catalase activity and MGO concentration in erythrocytes and plasma across four clinical groups of thyroid disease and a control group. The findings revealed different group-level patterns across the erythrocyte and plasma compartments. Erythrocyte catalase activity was significantly higher in all four patient groups than in controls, whereas plasma catalase activity was significantly higher only in the Hypo-nonAIT and euthyroid NNG groups. Erythrocyte MGO concentration was significantly lower than in controls in these same two groups, while plasma MGO concentration did not differ significantly among the study groups. However, most pairwise comparisons among the patient groups were not statistically significant; therefore, the findings do not establish distinct phenotype-specific biomarker profiles.

In the pooled patient group, the data did not support a significant monotonic relationship between catalase activity and MGO concentration within either blood compartment. In contrast, erythrocyte and plasma MGO concentrations were positively correlated, indicating partial rank-order concordance between the cellular and extracellular MGO pools. Nevertheless, these measurements should be interpreted separately because their between-group distributions differed. Owing to the cross-sectional design, the absence of covariate-adjusted analyses, and treatment heterogeneity, the findings do not establish causality or demonstrate the diagnostic or prognostic utility of the investigated biomarkers.

## 6. Limitations

The present findings should be interpreted in light of several limitations. First, the cross-sectional design allows between-group differences and correlations to be identified but does not permit causal relationships among thyroid disease, catalase activity, and methylglyoxal concentration to be established. In addition, this was a single-center study, which may limit the generalizability of the findings to other populations.

No a priori sample size calculation was performed, and the study was therefore not based on a prespecified statistical power target. Although age and sex distributions did not differ significantly among the study groups and BMI was reported descriptively for all groups, age, sex, and BMI were not included as covariates in adjusted models; therefore, residual confounding by demographic and anthropometric factors cannot be completely excluded. Smoking status and habitual iodine intake were not systematically assessed and could not be incorporated into adjusted analyses.

Levothyroxine treatment represents an additional source of heterogeneity within the hypothyroid groups. Although all participants in these groups met the biochemical criteria for hypothyroidism at enrollment, some were receiving levothyroxine whereas others were not. Treatment status was not used as a stratification variable because the resulting subgroups were small and markedly imbalanced and the study was not designed or powered for treatment-stratified analyses. Therefore, an independent effect of ongoing levothyroxine treatment on catalase activity or MGO concentration cannot be excluded.

Correlations calculated in the pooled patient group should be interpreted as overall associations across thyroid disease rather than phenotype-specific relationships, because pooling clinically heterogeneous thyroid phenotypes may obscure or modify associations present within individual groups. Finally, the biomarker panel was restricted to catalase activity and MGO concentration and did not include other potentially relevant components of oxidative and carbonyl metabolism, such as glyoxalase I, GSH, AGEs/sRAGE, AOPP, MDA, or inflammatory markers, which limits mechanistic interpretation of the observed associations.

## Figures and Tables

**Figure 1:**
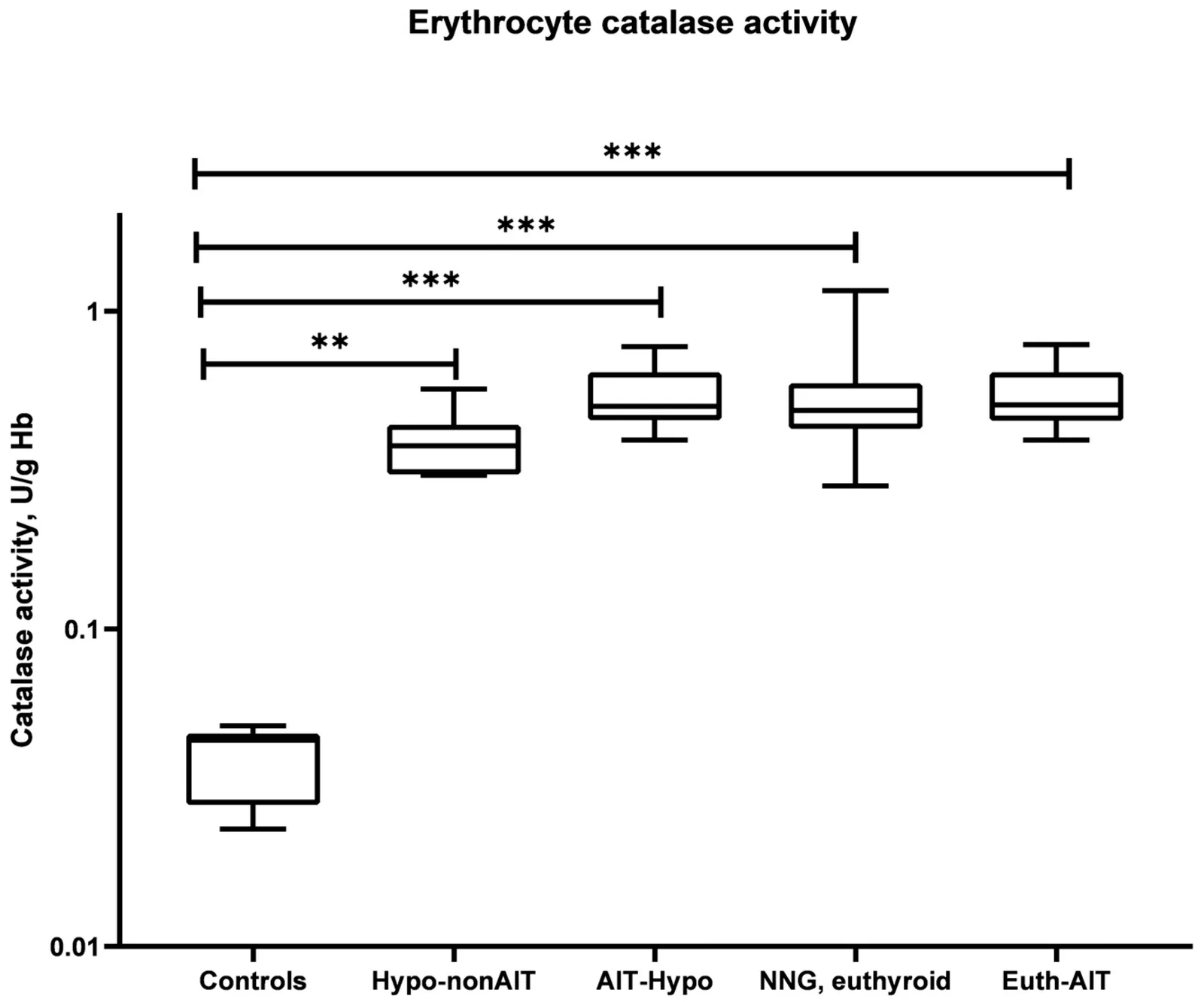
Distribution of erythrocyte catalase activity across the study groups. Statistically significant comparisons versus Controls are indicated by brackets and asterisks: ** *p* < 0.01 and *** *p* < 0.001; Dunn’s multiple-comparisons test following the Kruskal–Wallis test. Additional significant pairwise comparisons among patient groups are reported in the Results.

**Figure 2:**
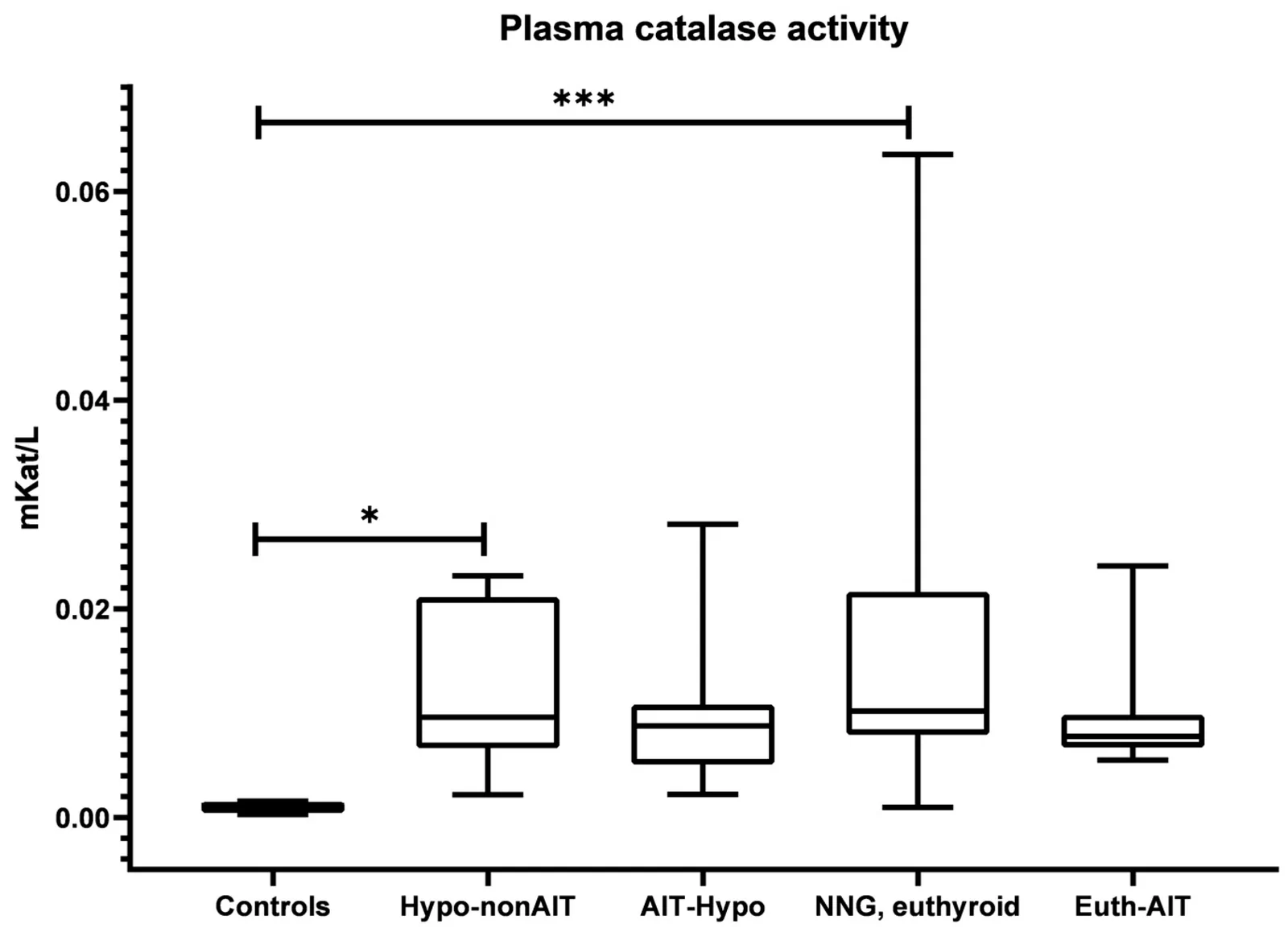
Distribution of plasma catalase activity across the study groups. Statistically significant comparisons versus Controls are indicated by brackets and asterisks: * *p* < 0.05 and *** *p* < 0.001; Dunn’s multiple-comparisons test following the Kruskal–Wallis test.

**Figure 3:**
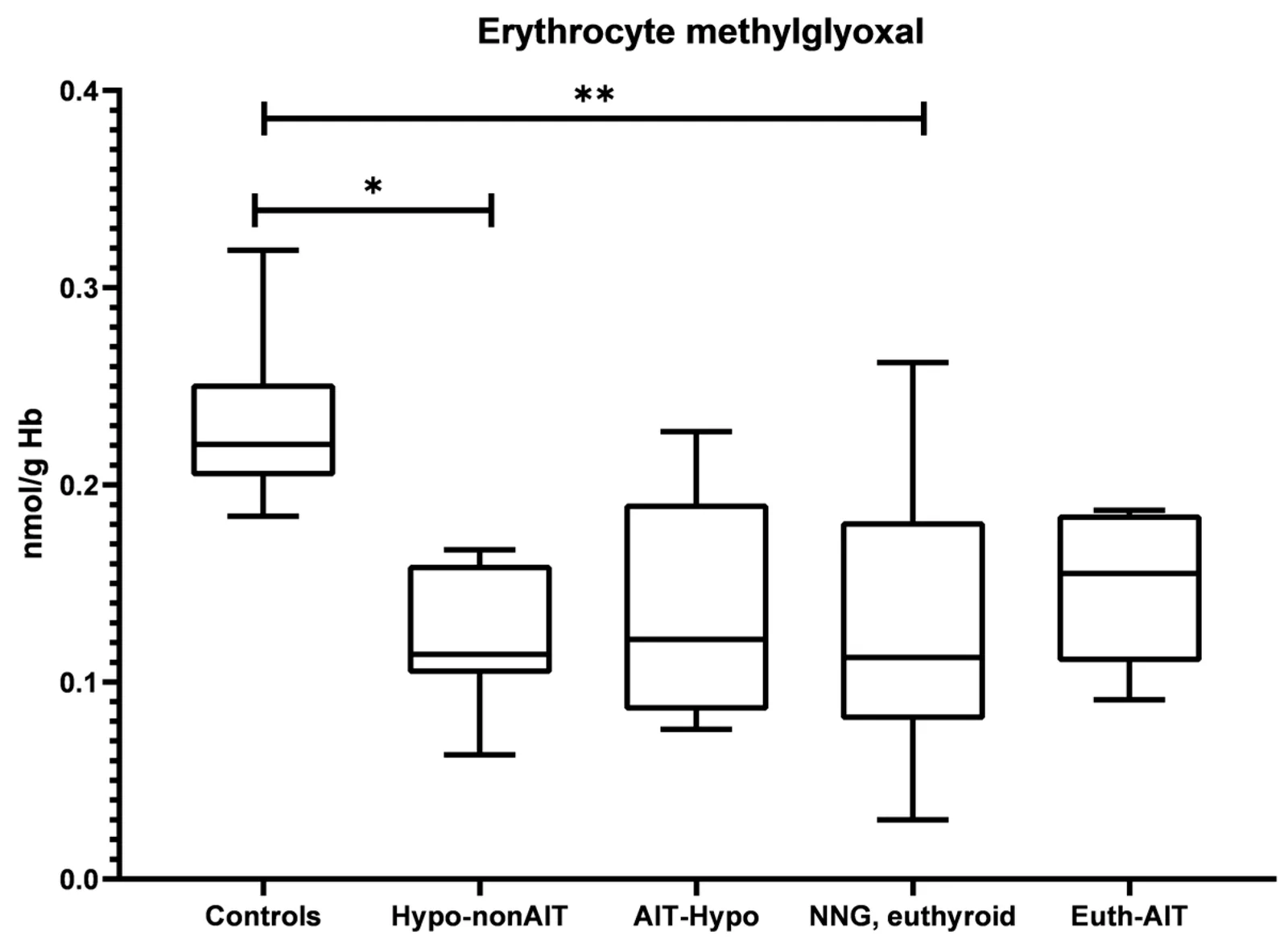
Distribution of erythrocyte MGO concentrations across the study groups. Statistically significant comparisons versus Controls are indicated by brackets and asterisks: * *p* < 0.05 and ** *p* < 0.01; Dunn’s multiple-comparisons test following the Kruskal–Wallis test.

**Figure 4:**
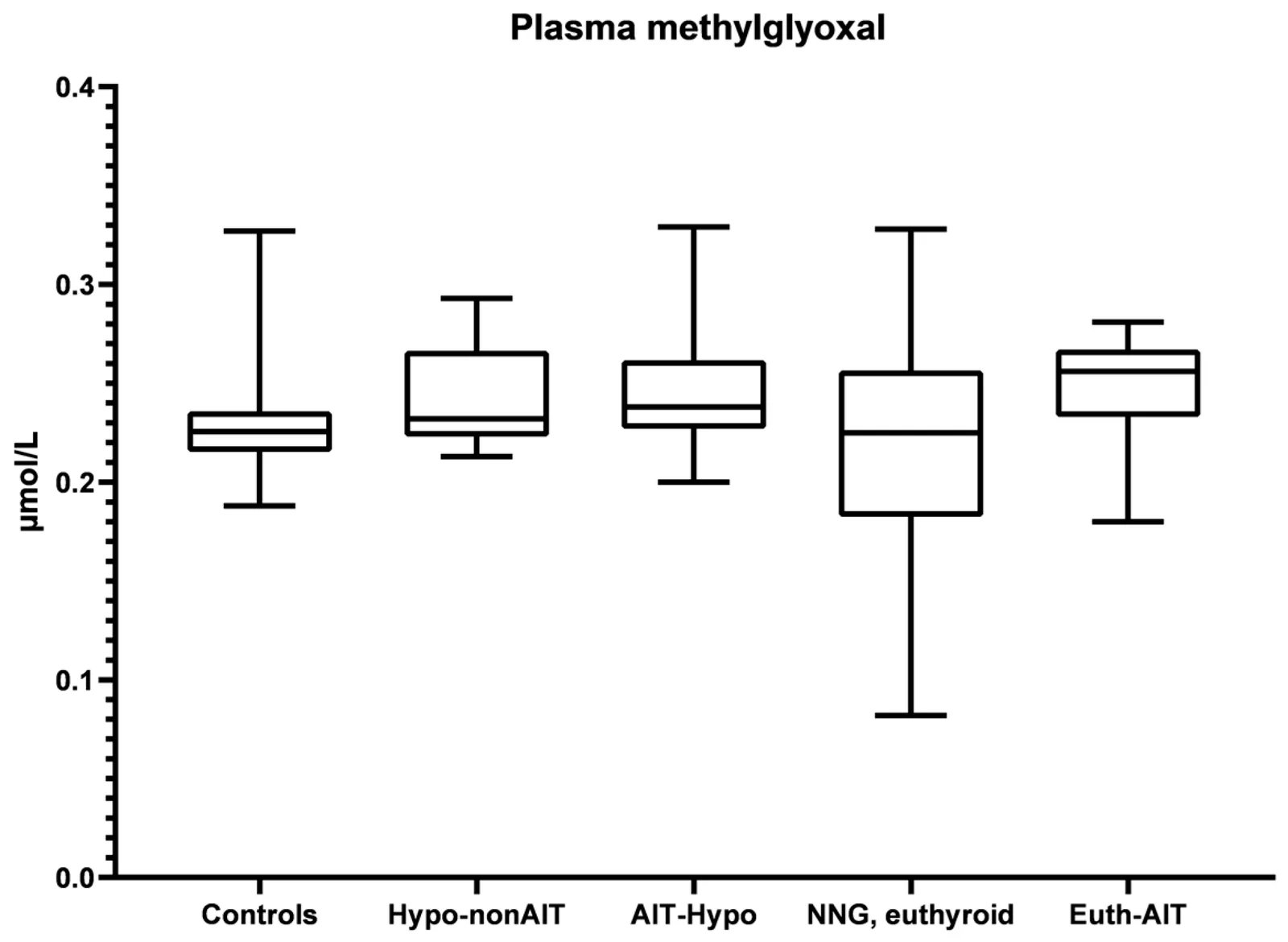
Distribution of plasma MGO concentrations across the study groups. No statistically significant between-group differences were identified (Kruskal–Wallis test), and none of the pairwise comparisons reached statistical significance in Dunn’s multiple-comparisons test.

**Figure 5:**
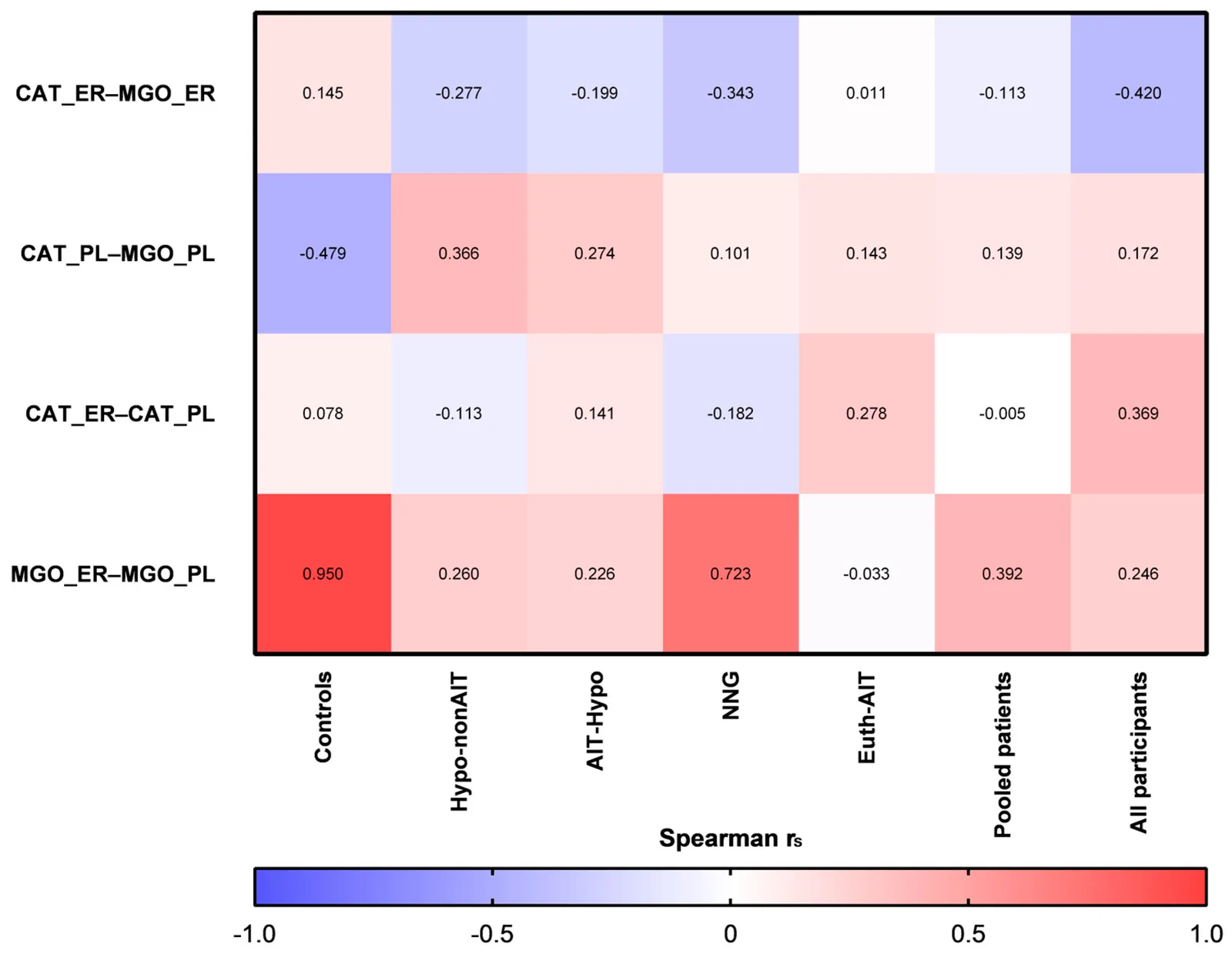
Heatmap of Spearman correlation coefficients among catalase activity and methylglyoxal concentrations in the control group, the four individual patient phenotypes (Hypo-nonAIT, AIT-Hypo, NNG, and Euth-AIT), the pooled group of patients with thyroid disease, and all participants. Cell values represent Spearman’s rank correlation coefficients (r_s_). Blue shading indicates negative correlations, red shading indicates positive correlations, and increasing color intensity reflects increasing correlation magnitude. CAT_ER, erythrocyte catalase activity; CAT_PL, plasma catalase activity; MGO_ER, erythrocyte methylglyoxal concentration; MGO_PL, plasma methylglyoxal concentration.

**Table 1:** Baseline demographic, clinical, laboratory, and treatment characteristics of the study groups.

Characteristic	Hypo-nonAIT (*n* = 31)	AIT-Hypo (*n* = 30)	NNG, Euthyroid (*n* = 42)	Euth-AIT (*n* = 37)	Control Group (*n* = 24)
Age, years, median [Q1–Q3]	41 [34–37]	43 [33–48]	39 [33–46]	44 [38–51]	40 [36–45]
Women, *n* (%)	25 (80.6%)	26 (86.7%)	32 (76.2%)	31 (83.8%)	16 (66.7%)
Men, *n* (%)	6 (19.4%)	4 (13.3%)	10 (23.8%)	6 (16.2%)	8 (33.3%)
BMI, kg/m^2^	29.3 [27.1–31.8]	26.2 [24.7–29.1]	27.9 [25.3–29.3]	26.2 [24.8–29.0]	25.8 [23.5–27.0]
TSH, mIU/L	7.95 [5.16–11.66]	8.52 [5.89–13.79]	2.19 [1.71–2.66]	2.00 [1.35–2.61]	1.85 [1.45–2.08]
fT4, pmol/L	12.3 [8.6–13.7]	11.8 [8.5–13.6]	16.3 [14.4–17.2]	14.2 [13.2–15.9]	15.7 [15.1–17.0]
anti-TPO, IU/mL	14.5 [8.7–20.0]	221.0 [162.7–415.3]	15.3 [9.8–18.7]	170.2 [81.0–369.4]	11.5 [8.3–16.2]
anti-TG, IU/mL	41.3 [31.1–67.0]	213.9 [112.9–269.0]	36.1 [22.9–57.5]	218.6 [119.5–408.4]	36.2 [24.0–45.2]
Subclinical hypothyroidism, *n* (%)	20 (64.5%)	18 (60.0%)	—	—	—
Overt hypothyroidism, *n* (%)	11 (35.5%)	12 (40.0%)	—	—	—
eGFR, mL/min/1.73 m^2^	91.4 [84.2–104.6]	92.5 [82.8–99.1]	101.1 [88.6–112.5]	96.9 [84.0–104.0]	100.5 [95.6–114.2]
L-T4 use, *n* (%)	22 (71.0%)	24 (80.0%)	0 (0.0%)	0 (0.0%)	0 (0.0%)
L-T4 dose, µg/kg/day	1.00 [0.73–1.28]	0.91 [0.73–1.18]	—	—	—
L-T4 treatment duration, months	39.5 [23.8–70.5]	46.5 [26.0–74.3]	—	—	—

Note. Continuous variables are presented as median [Q1–Q3] and categorical variables as *n* (%). L-T4 dose and treatment duration are presented for participants receiving levothyroxine only. A dash (—) indicates that the characteristic was not applicable. AIT, autoimmune thyroiditis; BMI, body mass index; TSH, thyroid-stimulating hormone; fT4, free thyroxine; anti-TPO, antibodies to thyroid peroxidase; anti-TG, antibodies to thyroglobulin; eGFR, estimated glomerular filtration rate; L-T4, levothyroxine.

**Table 2:** Erythrocyte catalase activity across the study groups.

Study Group	*n*	Median [Q1–Q3], U/g Hb	Min–Max, U/g Hb
Control group	24	0.04459 [0.02793–0.04685]	0.02342–0.04955
Hypo-nonAIT	31	0.3766 [0.3063–0.4387]	0.3045–0.5694
AIT-Hypo	30	0.5018 [0.4541–0.6423]	0.3928–0.7730
NNG, euthyroid	42	0.4878 [0.4266–0.5935]	0.2820–1.1590
Euth-AIT	37	0.5072 [0.4523–0.6423]	0.3928–0.7856

Note. Data are presented as median [Q1–Q3] and Min–Max. Hb, hemoglobin.

**Table 3:** Plasma catalase activity across the study groups.

Study Group	*n*	Median [Q1–Q3], mkat/L	Min–Max, mkat/L
Control group	24	0.001138 [0.000415–0.001485]	0.000270–0.001543
Hypo-nonAIT	31	0.009604 [0.006634–0.021118]	0.002160–0.023143
AIT-Hypo	30	0.008775 [0.005082–0.010810]	0.002199–0.028080
NNG, euthyroid	42	0.010183 [0.007926–0.021610]	0.000964–0.063527
Euth-AIT	37	0.007753 [0.006711–0.009836]	0.005477–0.024107

Note. Data are presented as median [Q1–Q3] and Min–Max.

**Table 4:** Erythrocyte MGO concentration across the study groups.

Study Group	*n*	Median [Q1–Q3], nmol/g Hb	Min–Max, nmol/g Hb
Control group	24	0.2205 [0.2043–0.2515]	0.1840–0.3190
Hypo-nonAIT	31	0.1140 [0.1040–0.1595]	0.0630–0.1670
AIT-Hypo	30	0.1215 [0.0855–0.1905]	0.0760–0.2270
NNG, euthyroid	42	0.1125 [0.0808–0.1815]	0.0300–0.2620
Euth-AIT	37	0.1550 [0.1100–0.1850]	0.0910–0.1870

Note. Data are presented as median [Q1–Q3] and Min–Max. Hb, hemoglobin.

**Table 5:** Plasma MGO concentration in the study groups.

Study Group	*n*	Median [Q1–Q3], µmol/L	Min–Max, µmol/L
Control group	24	0.2255 [0.2153–0.2358]	0.1880–0.3270
Hypo-nonAIT	31	0.2320 [0.2230–0.2665]	0.2130–0.2930
AIT-Hypo	30	0.2380 [0.2270–0.2618]	0.2000–0.3290
NNG, euthyroid	42	0.2250 [0.1825–0.2565]	0.0820–0.3280
Euth-AIT	37	0.2560 [0.2330–0.2670]	0.1800–0.2810

Note. Data are presented as median [Q1–Q3] and Min–Max.

**Table 6:** Correlations between catalase activity and methylglyoxal concentrations in erythrocytes and plasma.

Variable Pair	Control Group (*n* = 24), r_s_ (95% CI)	Holm-Adjusted *p*	Patients with Thyroid Disease (*n* = 140), r_s_ (95% CI)	Holm-Adjusted *p*	Combined Analytic Sample (*n* = 164), r_s_ (95% CI)	Holm-Adjusted *p*
Erythrocyte catalase activity–erythrocyte methylglyoxal concentration	0.145 (−0.275 to 0.518)	1.000	−0.113 (−0.274 to 0.054)	0.3689	−0.420 (−0.538 to −0.285)	<0.0001
Plasma catalase activity–plasma methylglyoxal concentration	−0.479 (−0.739 to −0.094)	0.0537	0.139 (−0.028 to 0.298)	0.3068	0.172 (0.020 to 0.317)	0.0274
Erythrocyte catalase activity–plasma catalase activity	0.078 (−0.336 to 0.466)	1.000	−0.005 (−0.171 to 0.161)	0.9519	0.369 (0.228 to 0.494)	<0.0001
Erythrocyte methylglyoxal concentration–plasma methylglyoxal concentration	0.950 (0.887 to 0.979)	<0.0001	0.392 (0.241 to 0.523)	<0.0001	0.246 (0.097 to 0.385)	0.0029

Note. r_s_, Spearman’s rank correlation coefficient; CI, confidence interval. Values are presented as r_s_ (95% CI). The confidence intervals were calculated using Fisher’s z transformation. Holm-adjusted *p* values account for the four predefined correlation tests performed within each analytic sample.

## Data Availability

The data presented in this study are available from the corresponding author upon reasonable request. The data are not publicly available due to privacy and ethical restrictions related to the protection of participants’ personal and clinical information.

## Supplementary Materials

The following supporting information can be downloaded at https://www.mdpi.com/article/10.3390/diseases14100357/s1. Supplementary Methods S1: Detailed catalase assay procedures, calculations, unit conversions, dilution-factor corrections, hemoglobin normalization, and analytical validation [33,34]; Supplementary Methods S2: Detailed methylglyoxal assay calibration, dilution-factor calculations, hemoglobin normalization, and analytical validation procedures [35].

## Abbreviations

AGEs	Advanced glycation end products
AIT	Autoimmune thyroiditis
anti-TG	Anti-thyroglobulin antibodies
anti-TPO	Anti-thyroid peroxidase antibodies
AOPP	Advanced oxidation protein products
BMI	Body mass index
CAT	Catalase activity
CI	Confidence interval
CV	Coefficient of variation
DF	Dilution factor
EC	Enzyme Commission
eGFR	Estimated glomerular filtration rate
fT3	Free triiodothyronine
fT4	Free thyroxine
GSH	Reduced glutathione
Hb	Hemoglobin
HT	Hashimoto thyroiditis
L-T4	Levothyroxine
LOD	Limit of detection
LOQ	Limit of quantification
MDA	Malondialdehyde
MGO	Methylglyoxal
NAC	N-acetyl-L-cysteine
SD	Standard deviation
sRAGE	Soluble receptor for advanced glycation end products
TSH	Thyroid-stimulating hormone
UV–Vis	Ultraviolet–visible
