# Supplementary material for: Catalase Activity and Methylglyoxal Concentration in Plasma and Erythrocytes Across Different Clinical Phenotypes of Thyroid Disease: A Cross-Sectional Study
Source: Diseases. 2026 Sep 26;14(10):357. doi: 10.3390/diseases14100357 (PMC13702957; doi:10.3390/diseases14100357)
Supplement: Supplementary file 1 [file diseases-14-00357-s001.zip › Supplementary_Methods_S1_Catalase.pdf]

Supplementary Materials

Catalase Activity and Methylglyoxal Concentration in Plasma and Erythrocytes Across Different Clinical Phenotypes of Thyroid Disease: A Cross-Sectional Study

Ernur Bekov, Olga Ponamareva, Ryszhan Bakirova, Vilen Molotov-Luchanskiy, Yelena Pozdnyakova

Supplementary Methods S1. Detailed Protocol, Calculations, and Analytical Validation Procedures for the Catalase Assay

S1.1. Analytical Principle

Catalase activity (EC 1.11.1.6) in plasma and erythrocyte hemolysate was measured spectrophotometrically using a modified ammonium molybdate method based on the procedures described by Koroliuk et al. and Góth [S1,S2]. The method is based on enzymatic decomposition of hydrogen peroxide (H_2O_2), followed by quantification of the residual H_2O_2 through formation of a colored complex with ammonium molybdate. The absorbance of the resulting complex is directly proportional to the residual H_2O_2 concentration and inversely proportional to catalase activity.

S1.2. Reagents and Sample Preparation

The assay reagents consisted of 50 mM potassium phosphate buffer (pH 7.0), freshly prepared 30 mM H_2O_2 working solution in potassium phosphate buffer, 4% ammonium molybdate solution, chilled 0.9% NaCl solution, and chilled distilled water. The H_2O_2 working solution was prepared immediately before each assay from a hydrogen peroxide stock solution, with correction for its actual concentration.

For catalase determination, plasma was initially analyzed without dilution (dilution factor [DF] = 1). The validated working range of the plasma catalase assay was 0.01–20.0 U/mL, corresponding to 0.000167–0.333 mkat/L. If the measured absorbance exceeded the validated analytical range, an aliquot of plasma was diluted with 50 mM potassium phosphate buffer (pH 7.0) to bring the measurement within the working range. The catalase activity obtained after dilution was multiplied by the actual dilution factor used for that sample.

For erythrocyte catalase determination, packed erythrocytes remaining after plasma separation were washed three times with chilled 0.9% NaCl solution. After each wash, the erythrocyte suspension was centrifuged at 3000 × g for 10 min at 4 °C and the supernatant was removed completely. One volume of washed packed erythrocytes was mixed with nine volumes of chilled distilled water to produce a 1:10 erythrocyte hemolysate. The mixture was thoroughly mixed and maintained at 4 °C until hypotonic hemolysis was complete. If necessary, the hemolysate was further diluted with potassium phosphate buffer or distilled water to bring the absorbance within the linear range of the assay, and the additional dilution factor was included in the calculation. A separate aliquot of the same hemolysate was used for hemoglobin determination.

S1.3. Enzymatic Reaction, Control Reaction, and Reagent Blank

For each test reaction, 1.0 mL of freshly prepared 30 mM H_2O_2 solution was mixed with 0.1 mL of plasma or erythrocyte hemolysate. The mixture was immediately mixed and incubated for 60 s at 25 °C. The reaction was then terminated by adding 1.0 mL of 4% ammonium molybdate solution, resulting in a final reaction volume of 2.1 mL.

A control reaction was prepared in parallel for each biological sample. For the control reaction, 1.0 mL of 30 mM H_2O_2 solution was first mixed with 1.0 mL of 4% ammonium molybdate solution, followed by

addition of 0.1 mL of plasma or erythrocyte hemolysate. Because ammonium molybdate was added before the biological sample, enzymatic decomposition of H_2O_2 was prevented. The control reaction therefore represented the initial H_2O_2 concentration while also accounting for the intrinsic absorbance of the biological sample.

A reagent blank contained all assay reagents but no biological material. For plasma measurements, 0.1 mL of potassium phosphate buffer was added instead of plasma; for erythrocyte measurements, 0.1 mL of distilled water was added instead of hemolysate. Absorbance of both the test and control reactions was measured against the corresponding reagent blank.

Before spectrophotometric measurement, test and control mixtures were diluted 20-fold with distilled water to bring residual H_2O_2 concentrations within the linear range of the method. Absorbance was measured at 410 nm in a 1-cm optical path-length cuvette using a PD-303UV digital UV–Vis spectrophotometer (APEL, Japan).

S1.4. Calibration of Residual H_2O_2

Residual H_2O_2 was quantified using a calibration curve constructed from at least five H_2O_2 standard solutions. The calibration range was 0.5–20.0 mM H_2O_2 when expressed relative to the original reaction mixture before dilution, corresponding to 0.025–1.0 mM in the solution subjected to spectrophotometric measurement. Each standard was processed under conditions identical to those used for the study samples, and absorbance was measured at 410 nm.

The calibration curve was described by the linear regression equation:

$$A_{410} = a \times C(H_2O_2) + b$$

where A_{410} is the absorbance at 410 nm, $C(H_2O_2)$ is the H_2O_2 concentration, a is the slope, and b is the intercept. Residual H_2O_2 concentration was calculated as:

$$C(H_2O_2) = (A_{410} - b) / a$$

The amount of H_2O_2 decomposed during the 60-s incubation was calculated from the difference between the control and test reactions:

$$\Delta C(H_2O_2) = C_0 - C_t$$

where C_0 is the H_2O_2 concentration in the control reaction and C_t is the residual H_2O_2 concentration in the test reaction.

S1.5. Calculation of Catalase Activity

Catalase activity (CAT) was calculated as:

$$CAT = [(C_0 - C_t) \times Vfinal \times DF] / (t \times Vsample)$$

where Vfinal = 2.1 mL, Vsample = 0.1 mL, t = 1 min, and DF is the additional sample dilution factor. Under these assay conditions:

$$CAT = (C_0 - C_t) \times 21 \times DF$$

One unit of catalase activity was defined as the amount of enzyme decomposing 1 μmol of H_2O_2 per minute under the specified assay conditions.

S1.6. Calculation of Plasma Catalase Activity

Plasma catalase activity was initially expressed in U/mL and converted to mkat/L according to:

$$CATplasma\ (mkat/L) = CATplasma\ (U/mL) / 60$$

Thus, 1 U/mL corresponded to 0.01667 mkat/L.

S1.7. Normalization of Erythrocyte Catalase Activity to Hemoglobin

Erythrocyte catalase activity was normalized to hemoglobin concentration and expressed as U/g Hb:

CATerythrocytes (U/g Hb) = CAThemolysate (U/mL) × 1000 / Hbhemolysate (g/L)

The initial 1:10 dilution used to prepare the erythrocyte hemolysate was not included separately in this calculation because catalase activity and hemoglobin concentration were measured in the same hemolysate and were therefore affected equally by this dilution. Hemoglobin concentration was determined in a separate aliquot of the same hemolysate using the cyanmethemoglobin (Drabkin) method with a Human assay kit (Human GmbH, Germany) at 540 nm.

S1.8. Analytical Quality Control and Performance

All biological samples were analyzed in duplicate, and the arithmetic mean of the two measurements was used for statistical analysis. If the relative difference between duplicate measurements exceeded 10%, the sample was reanalyzed.

Linearity of residual H_2O_2 determination was evaluated by regression analysis using a series of standard solutions with at least two replicate measurements at each calibration level. Dilutional linearity was assessed by serial dilution of pooled plasma and erythrocyte hemolysate samples followed by back-calculation using the corresponding dilution factors.

Within-run precision was assessed by repeated measurement of a control sample within a single analytical run, whereas between-run precision was evaluated using measurements performed on different days. The within-run coefficient of variation (CV) was 4.8% and the between-run CV was 7.2%. Deviations after serial dilution and back-calculation ranged from −8% to +8%.

The validated working range of the plasma catalase assay was 0.01–20.0 U/mL, corresponding to 0.000167–0.333 mkat/L. The observed plasma catalase activities ranged from 0.0162 to 3.8118 U/mL (0.000270–0.063530 mkat/L) and therefore fell within the validated analytical range.

Abbreviations

CAT, catalase activity; CV, coefficient of variation; DF, dilution factor; Hb, hemoglobin; H_2O_2, hydrogen peroxide.

References

S1. Koroliuk, M.A.; Ivanova, L.I.; Maiorova, I.G.; Tokarev, V.E. A method of determining catalase activity. Lab. Delo 1988, 1, 16–19. (In Russian).

S2. Góth, L. A simple method for determination of serum catalase activity and revision of reference range. Clin. Chim. Acta 1991, 196, 143–151. https://doi.org/10.1016/0009-8981(91)90067-M.
