# Supplementary material for: Catalase Activity and Methylglyoxal Concentration in Plasma and Erythrocytes Across Different Clinical Phenotypes of Thyroid Disease: A Cross-Sectional Study
Source: Diseases. 2026 Sep 26;14(10):357. doi: 10.3390/diseases14100357 (PMC13702957; doi:10.3390/diseases14100357)
Supplement: Supplementary file 1 [file diseases-14-00357-s001.zip › Supplementary_Methods_S2_MGO.pdf]

Supplementary Materials

Catalase Activity and Methylglyoxal Concentration in Plasma and Erythrocytes Across Different Clinical Phenotypes of Thyroid Disease: A Cross-Sectional Study

Ernur Bekov, Olga Ponamareva, Ryszhan Bakirova, Vilen Molotov-Luchanskiy, Yelena Pozdnyakova

Supplementary Methods S2. Detailed Protocol, Calculations, Calibration, and Analytical Validation Procedures for MGO Determination

S2.1. Analytical Principle

Methylglyoxal (MGO) concentrations in plasma and erythrocyte hemolysate were measured spectrophotometrically after derivatization with N-acetyl-L-cysteine (NAC) using a substantially modified version of the method described by Wild et al. [S1], adapted and validated for determination of MGO in the micromolar range in plasma and erythrocyte hemolysate. The method is based on the reaction of MGO with NAC to form N-α-acetyl-S-(1-hydroxy-2-oxoprop-1-yl)cysteine, which has an absorption maximum at 288 nm.

S2.2. Reagents

The following reagents were used: 0.6 M perchloric acid solution; 2 M potassium carbonate solution; 100 mM phosphate buffer (pH 7.0); freshly prepared 500 mM NAC solution in 100 mM phosphate buffer (pH 7.0); MGO standard solution of known concentration; chilled 0.9% NaCl solution; and chilled distilled water. The NAC solution was prepared immediately before the assay, maintained at 4 °C until use, and protected from prolonged exposure to light.

S2.3. Plasma Sample Preparation

Plasma was mixed with 0.6 M perchloric acid at a 1:1 ratio (v/v). The mixture was mixed thoroughly, incubated on ice for 10 min, and centrifuged at 10,000 × g for 10 min at 4 °C. The resulting supernatant was transferred to a clean tube and neutralized to pH 7.0 with 2 M K_2CO_3 solution. The actual volume of K_2CO_3 solution used to neutralize each sample was recorded and included in the calculation of the overall dilution factor.

After neutralization, the samples were incubated on ice for 10 min and centrifuged again at 10,000 × g for 10 min at 4 °C. The resulting potassium perchlorate precipitate was removed, and the clear supernatant was used to determine plasma MGO concentration.

S2.4. Preparation of Erythrocyte Hemolysate

Erythrocytes were washed three times with chilled 0.9% NaCl solution. After each wash, the samples were centrifuged at 3,000 × g for 10 min at 4 °C, and the supernatant was completely removed. One volume of washed packed erythrocytes was mixed with nine volumes of chilled distilled water. The resulting suspension was mixed thoroughly and maintained at 4 °C until hypotonic hemolysis was complete.

The resulting hemolysate was mixed with 0.6 M perchloric acid at a 1:1 ratio (v/v). The mixture was incubated on ice for 10 min and centrifuged at 10,000 × g for 10 min at 4 °C. The supernatant was transferred to a clean tube and neutralized to pH 7.0 with 2 M K_2CO_3 solution. The actual volume of K_2CO_3 solution used was recorded.

After neutralization, the sample was incubated on ice for 10 min and centrifuged again at 10,000 × g for 10 min at 4 °C. The clear supernatant was used to determine erythrocyte MGO concentration. A separate

aliquot of the original hemolysate, collected before deproteinization with perchloric acid, was used to measure hemoglobin concentration.

S2.5. Derivatization Reaction

The total reaction volume was 1.0 mL. The following components were added sequentially to the reaction tube: 930 µL of 100 mM phosphate buffer (pH 7.0), 50 µL of deproteinized and neutralized sample, and 20 µL of freshly prepared 500 mM NAC solution. The final concentration of NAC in the reaction mixture was 10 mM.

After addition of NAC, the mixture was mixed immediately and incubated for 10 min at 25 °C. Absorbance was measured at 288 nm in a quartz cuvette with a 1-cm optical path length using a PD-303UV digital UV–Vis spectrophotometer (APEL, Japan).

S2.6. Matrix Control and Reagent Blank

A matrix control was prepared in parallel for each biological sample and contained 950 µL of 100 mM phosphate buffer (pH 7.0) and 50 µL of the prepared biological sample. The matrix control contained the same volume of biological material as the test reaction but did not contain NAC; the volume of NAC solution was replaced with an equal volume of phosphate buffer. The matrix control was used to correct for the intrinsic absorbance of plasma or erythrocyte hemolysate components at 288 nm.

The reagent blank contained 980 µL of 100 mM phosphate buffer (pH 7.0) and 20 µL of 500 mM NAC solution and contained no biological material. Absorbance was measured against 100 mM phosphate buffer (pH 7.0). For each biological sample, corrected absorbance was calculated by subtracting both the corresponding matrix-control and reagent-blank absorbance from the absorbance of the complete derivatization reaction.

Corrected absorbance was calculated as follows:

$A_{288,corrected} = A_{288,reaction} - A_{288,matrix} - A_{288,reagent\ blank}$

where $A_{288,reaction}$ is the absorbance of the complete derivatization reaction, $A_{288,matrix}$ is the absorbance of the corresponding biological sample without NAC, and $A_{288,reagent\ blank}$ is the absorbance of NAC in phosphate buffer without biological material.

S2.7. Construction of the Calibration Curve

The calibration curve was constructed using a zero calibrator and at least five MGO standard solutions. Standard concentrations were selected so that their analytical signals covered the range observed for the study samples. Concentrations were calculated within the linear portion of the calibration curve. The overall dilution factor was included when recalculating results for the original plasma or erythrocyte hemolysate.

Each calibration sample contained 930 µL of 100 mM phosphate buffer (pH 7.0), 50 µL of MGO standard solution, and 20 µL of 500 mM NAC solution. Calibration samples were mixed, incubated for 10 min at 25 °C, and measured at 288 nm.

The calibration curve was described by the following linear regression equation:

$A_{288,corrected} = a \times C(MGO) + b$

where $A_{288,corrected}$ is the corrected absorbance at 288 nm; C(MGO) is the MGO concentration in the reaction mixture; a is the slope of the calibration curve; and b is the intercept of the linear regression equation. The MGO concentration in the reaction mixture was calculated as follows:

$C(MGO)_{reaction} = [A_{288,corrected} - b] / a$

A separate calibration curve was constructed for each analytical run. Standards were analyzed in at least two technical replicates, and the mean absorbance value for each calibration level was used to construct the calibration curve.

S2.8. Calculation of the Overall Plasma Dilution Factor

The dilution factor resulting from mixing plasma with 0.6 M perchloric acid at a 1:1 ratio was:

DFdeproteinization = 2

The dilution factor resulting from neutralization was calculated as follows:

DFneutralization = [Vsupernatant + V(K_2CO_3)] / Vsupernatant

where Vsupernatant is the supernatant volume before neutralization and V(K_2CO_3) is the volume of 2 M K_2CO_3 solution used to achieve pH 7.0.

The dilution factor introduced during the reaction was:

DFreaction = Vreaction / Vsample

At a total reaction volume of 1.0 mL and a sample volume of 0.05 mL:

DFreaction = 1.0 / 0.05 = 20

The overall plasma dilution factor was calculated as follows:

DFplasma = DFdeproteinization × DFneutralization × DFreaction × DFadd

where DFadd is the additional sample dilution factor, when applicable. Therefore:

DFplasma = 2 × DFneutralization × 20 × DFadd

S2.9. Calculation of Plasma MGO Concentration

The MGO concentration in the original plasma sample was calculated as follows:

C(MGO)plasma = C(MGO)reaction × DFplasma

Results were expressed in µmol/L of plasma.

S2.10. Calculation of MGO Concentration in Erythrocyte Hemolysate

To calculate hemoglobin-normalized erythrocyte MGO concentration, the MGO concentration in the prepared erythrocyte hemolysate was first determined. The overall dilution factor applied after preparation of the hemolysate was calculated as follows:

DFhemolysate = DFdeproteinization × DFneutralization × DFreaction × DFadd

Therefore:

DFhemolysate = 2 × DFneutralization × 20 × DFadd

The initial 1:10 dilution of packed erythrocytes used to prepare the hemolysate was not included in the calculation of the result expressed in nmol/g Hb because MGO and hemoglobin concentrations were measured in the same hemolysate. The initial dilution of packed erythrocytes affected MGO and Hb concentrations equally and therefore canceled when their ratio was calculated.

The MGO concentration in the hemolysate was calculated as follows:

C(MGO)hemolysate = C(MGO)reaction × DFhemolysate

The result was expressed in µmol/L of hemolysate.

S2.11. Normalization of Erythrocyte MGO Concentration to Hemoglobin

Erythrocyte MGO concentration was expressed in nmol/g Hb. The calculation was performed using the following equation:

$$MGO_{erythrocytes}\ (\text{nmol/g Hb}) = [C(MGO)_{hemolysate}\ (\mu\text{mol/L}) \times 1{,}000] / Hb_{hemolysate}\ (\text{g/L})$$

The factor of 1,000 was used to convert micromoles to nanomoles. Hemoglobin concentration was measured in an aliquot of the original hemolysate collected before deproteinization, using the cyanmethemoglobin (Drabkin) method, a Human assay kit (Human GmbH, Germany), and a wavelength of 540 nm.

S2.12. Technical Replicates

All biological samples were assayed in duplicate. The arithmetic mean of the two measurements was used for subsequent statistical analysis. The relative difference between duplicate measurements was calculated as follows:

$$\text{Difference (\%)} = |X_1 - X_2| / [(X_1 + X_2) / 2] \times 100$$

If the difference between duplicate measurements exceeded the predefined acceptance limit of 10%, the sample was reanalyzed.

S2.13. Assessment of Linearity

The linearity of the analytical response was evaluated by regression analysis using at least five nonzero calibration levels and a zero calibrator. At least two replicate measurements were performed at each calibration level. The linear regression equation, coefficient of determination, and residual distribution were evaluated. The actual R^2 value was reported only on the basis of experimentally obtained calibration results. The working range was defined as the concentration range over which linearity, acceptable precision, and accuracy had been demonstrated.

S2.14. Assessment of Within-Run and Between-Run Precision

Within-run precision was evaluated by repeated analysis of control samples within a single analytical run. Between-run precision was evaluated by analyzing the same control samples on different days. The coefficient of variation was calculated using the following equation:

$$\text{CV (\%)} = SD / \bar{x} \times 100$$

where SD is the standard deviation of repeated measurements and $\bar{x}$ is the arithmetic mean.

S2.15. Accuracy Assessment by Spike-and-Recovery

The accuracy of MGO determination was evaluated using spike-and-recovery experiments. Known amounts of MGO standard solution were added to plasma and erythrocyte hemolysate samples. Spiked and unspiked samples underwent the complete deproteinization, neutralization, and derivatization procedure. Recovery of the added MGO was calculated as follows:

$$\text{Recovery (\%)} = [(C_{spiked} - C_{original}) / C_{added}] \times 100$$

where C_{spiked} is the measured MGO concentration in the sample after addition of the standard; $C_{original}$ is the initial MGO concentration in the unspiked sample; and C_{added} is the known MGO concentration added to the sample. Recovery was evaluated separately for plasma and erythrocyte hemolysate.

S2.16. Analytical Performance Characteristics

The method was validated separately for plasma and erythrocyte hemolysate. For plasma, the limit of detection (LOD) was 0.002 µmol/L, the limit of quantification (LOQ) was 0.005 µmol/L, the within-run CV was 5.2%, the between-run CV was 7.8%, recovery of added MGO ranged from 93% to 107%, and the matrix effect was −6.5%.

For erythrocyte hemolysate, the LOD was 0.003 µmol/L, the LOQ was 0.008 µmol/L, the within-run CV was 6.1%, the between-run CV was 8.9%, recovery of added MGO ranged from 90% to 105%, and the matrix effect was +8.2%.

S2.17. Assessment of Biological Matrix Effects

The effects of the plasma and erythrocyte hemolysate matrices were evaluated based on analyte recovery and the results of serial sample dilution. To assess dilutional parallelism, each sample was serially diluted. The measured concentration obtained after each dilution was multiplied by the corresponding dilution factor and compared with the concentration measured in the original sample. Comparable dilution-corrected concentrations across serial dilutions indicated the absence of substantial nonlinear matrix interference within the investigated range.

S2.18. Determination of the Limit of Detection and Limit of Quantification

The limit of detection was calculated as follows:

$LOD = 3.3 \times \sigma / a$

where σ is the standard deviation of the analytical signal obtained from blank or low-concentration samples and a is the slope of the calibration curve.

The limit of quantification was calculated as follows:

$LOQ = 10 \times \sigma / a$

LOD and LOQ values were expressed in the same concentration units used to construct the calibration curve.

Abbreviations

CV, coefficient of variation; DF, dilution factor; Hb, hemoglobin; LOD, limit of detection; LOQ, limit of quantification; MGO, methylglyoxal; NAC, N-acetyl-L-cysteine.

References

S1. Wild, R.; Ooi, L.; Srikanth, V.; Münch, G. A quick, convenient and economical method for the reliable determination of methylglyoxal in millimolar concentrations: The N-acetyl-L-cysteine assay. Anal. Bioanal. Chem. 2012, 403, 2577–2581. https://doi.org/10.1007/s00216-012-6086-4.
